# Long-Term Projections of Cancer Incidence and Mortality in Japan and Decomposition Analysis of Changes in Cancer Burden, 2020–2054: An Empirical Validation Approach

**DOI:** 10.3390/cancers14246076

**Published:** 2022-12-09

**Authors:** Phuong The Nguyen, Eiko Saito, Kota Katanoda

**Affiliations:** 1 Graduate School of Public Health, St. Luke’s International University, Tokyo 104-0045, Japan; 2 Division of Surveillance and Policy Evaluation, National Cancer Center Institute for Cancer Control, Tokyo 104-0045, Japan; 3 Institute for Global Health Policy Research, National Center for Global Health and Medicine, Tokyo 162-8655, Japan

**Keywords:** empirical validation, long-term projection, age–period–cohort, cancer burden, prostate, female breast, lung, colorectal

## Abstract

**Simple Summary:**

This is the first comprehensive picture of cancer incidence and mortality projections in Japan, using the most up-to-date and nationally representative data to the best of our knowledge. We applied empirically validated statistical models to project cancer incidence and mortality from 2020 to 2054 for all 22 cancer sites and in both genders. Additionally, we provided the decomposition analysis of changes in cancer cases and deaths over 2020–2054 attributable to population aging, population decline, and changes in the population’s risk of being diagnosed with or dying from cancer. We projected an increase in new cancer cases but a decrease in cancer deaths in both genders. Prostate and female breast cancers would be the leading cancer burdens among the Japanese population in 2050–2054, together with colorectal and lung cancers. Our findings warrant greater efforts in cancer control programs, specifically in enhancing cancer screening and controlling cancer risk factors in Japan.

**Abstract:**

Purpose: The aim of this study was to project new cancer cases/deaths forward to 2054, and decompose changes in cancer cases/deaths to assess the impact of demographic transitions on cancer burden. Methods: We collected data on cancer cases/deaths up to 2019, empirically validated the projection performance of multiple statistical models, and selected optimal models by applying time series cross-validation. Results: We showed an increasing number of new cancer cases but decreasing number of cancer deaths in both genders, with a large burden attributed to population aging. We observed the increasing incidence rates in most cancer sites but reducing rates in some infection-associated cancers, including stomach and liver cancers. Colorectal and lung cancers were projected to remain as leading cancer burdens of both incidence and mortality in Japan over 2020–2054, while prostate and female breast cancers would be the leading incidence burdens among men and women, respectively. Conclusions: Findings from decomposition analysis require more supportive interventions for reducing mortality and improving the quality of life of Japanese elders. We emphasize the important role of governments and policymakers in reforming policies for controlling cancer risk factors, including oncogenic infections. The rapid increase and continued presence of those cancer burdens associated with modifiable risk factors warrant greater efforts in cancer control programs, specifically in enhancing cancer screening and controlling cancer risk factors in Japan.

## 1. Introduction

Since cancer is the leading cause of death worldwide (accounting for 10 million deaths in 2020) [1], long-term projections of cancer burdens (incidence and mortality) are critical for allocating national healthcare resources, evaluating the interventions implemented, and planning future programs. As cancer occurs mainly in older adults (those aged ≥65 years), it is projected that a worldwide demographic transition (i.e., population aging and increasing) will lead to a substantial increase in the cancer burdens on public health in the future [2,3]. Specifically, the International Agency for Research on Cancer forecasted a global growth of 10.9 million cancer cases and 6.3 million cancer deaths by 2040 due to demographic changes [4]. Country-specific projections of cancer, however, should reflect both future changes in demographic components (i.e., population structure and population size) and epidemiological components (i.e., population risk of being diagnosed with or dying from cancer). This information has been in great demand as it can support policymakers and governments in demand-based resource allocations for patient-centered healthcare and evidence-based cancer policy transformation.

In Japan, cancer has been the number one cause of death since 1981 [5], accounting for one-third of total deaths in the country with the highest life expectancy among the Group of Seven nations [6]. Recent research has shown a leveling off of the age-standardized incidence rate (ASIR) and a decrease in the age-standardized mortality rate (ASMR) in all-site-combined cancers in Japan [7]. Nevertheless, these findings cannot suggest a reduction in the future cancer burden as age-standardized rates (ASR) adjust to a common standard population and do not factor in the influence of demographic changes. Notably, Japan has a super-aging society (proportion of older adults in 2020 of 28.8%) [8], but a decreasing population (as the fertility rate is lower than the death rate) [9] and uniquely short “baby booms” (only 1947–1949 and 1971–1974) [10]; thus, it has been challenged by complicated issues in economic, society, and healthcare sustainability. These demographic transitions, in combination with the complex mixture of reductions in cancer risk factor exposures (e.g., reduced smoking prevalence and infections) and improvements in cancer control (e.g., cancer diagnosis and treatment), require an investigation of long-term projections of cancer cases/deaths in Japan [11,12,13]. Additionally, the decomposition of changes in cancer cases/deaths attributable to demographic components (i.e., population aging and population decline) and epidemiological components (i.e., the shift in risk factor exposures, and improvements in cancer control, diagnosis, and treatment) further support evaluating the impact of demographic transitions on cancer burdens in Japan [14].

Among the different methods for projecting cancer burdens (e.g., microsimulation models, autoregression models, piece-wise linear regression models, generalized linear models, and Bayesian models), age–period–cohort (APC) models consider all components of age, calendar period, and birth cohort and allow for the incorporation of demographic transitions in population aging and growth [15,16]. APC models have been widely adopted for long-term cancer burden’s projections of both common and rare cancers in numerous countries, including the US [17,18], UK [19,20], Canada [21], Australia [22], Switzerland [23], and the Nordic countries [24]. Nevertheless, the flexibility of the APC models, with dozens of different approaches, has posed difficulties in selecting the most appropriate model for a specific context, which usually requires an empirical comparison for decision-making [25]. Despite the important role of cancer burden’s projections, none of the previous studies validated a statistical model and produced long-term projections for all cancer sites in Japan, although one model (age–period interaction model with spline smoothing) was validated for short-term cancer projections [26].

Here, we provide long-term projections of cancer incidence and mortality for all cancer sites in Japan based on empirically validated statistical models. We then present a decomposition analysis of changes in cancer cases and deaths over the study period to further assess the burden of population aging on cancer incidence and mortality in Japan.

## 2. Materials and Methods

**Data sources:** We collected the number of cases and deaths by gender for 22 cancer sites over the period 1975–2019 from the Cancer Statistics in Japan (CSJ) [27,28], and classified them into the major, sub-major, and other cancer groups based on leading cancers in Japan (either in incidence or mortality, and in males or females), similar to previous studies [7]. Detailed gender-specific information on the included cancer sites, classifications, and International Classification of Diseases codes are presented in Supplementary Table S1. In total, we gathered 39 gender–site combinations including 17 sites for males and females plus five gender-specific sites (17 × 2 + 5). We used estimated population data (1975–2019), also from CSJ, and the projected population (2020–2054) from the Institute of Population and Social Security with the medium-fertility variant and medium-mortality rate assumptions [29]. The population data of Japan reveal an aging population with increasing proportions of older people, and a decreasing total population (Supplementary Figure S1).

**Data processing:** We aggregated data into 5-year age groups and 5-year calendar periods to have statistically sufficient numbers of cancer cases and deaths in younger age groups or rare cancer sites. Specifically, we generated 18 5-year age groups (0–5 up to 85+ years) in nine periods (from 1975–1979 to 2015–2019) for cancer cases and deaths, and in 16 periods (from 1975–1979 to 2020–2054) for population data. The numbers of breast cancer cases in 1975–2002 included carcinoma in situ (CIS), and they were incomparable with those excluding CIS in 2003–2019. We, thus, estimated the number of cases excluded CIS in 1975–2002 using the mean ratio of cases excluded CIS per case included CIS for each five-year age group in 2003–2019.

**Projection models:** In this study, we investigated APC models, including classical and several modified versions, for application to cancer projection. These models have been developed on the basis of 5-year age groups, 5-year periods, and birth cohorts (synthetically constructed by subtracting age from period), and they have been widely applied for projecting cancer incidence and mortality [17,20,24,30]. The generalized APC can be described as

(1) $$\lambda_{ap}=f\left(A_{a}+D_{p}+P_{p}+C_{c}\right),\;$$

where $\lambda_{ap}$ is the rate (incidence or mortality) in age group *a* and calendar period *p*, $A_{a}$ is the age component of age group *a*, $P_{p}$ is the nonlinear period component of period p, and $C_{c}$ is the nonlinear cohort component of cohort c=p−a. The link function f(x) is either an exponential function in the classical model [31] or a power function, e.g., $f\left(x\right)=x^{w}$, in modified models, presumedly aimed at leveling off exponential increasing/decreasing trends [32,33]. The drift parameter D is employed to deal with collinearity among age, period, and cohort (so-called identifiability problem) and estimate the common linear effect between birth cohort and calendar period [31]. Projections can be produced using either D as the average linear trend of the whole observed period, or

(2) $$D_{last}=D-P_{p-2}\;$$

as the slope of the most recent two periods (where $P_{p-2}$ is the last deviation from this), or an algorithm for automatically selecting D or $D_{last}$ by testing whether the curvature in trends over time $\hat{S}$ is statistically significant [25], using the model

(3) $$\lambda_{ap}=f\left(A_{a}+D_{p}+S_{p}^{2}+C_{c}\right).\;$$

Additionally, some attenuation may be adopted to the drift according to the belief that observed trends would not indefinitely continue but eventually fade off [17,24,30], e.g., geometrically attenuating by *k*% every year using the formula

(4) $$1-{\left(1-k/100\right)}^{n},\;$$

where *n* is the number of calendar years [19,20]. In total, we generated 90 model variants from six potential link functions (Poisson, Power-3, Power-4, Power-5, Power-6, and Power-7), with three options of using a linear trend (D, $D_{last}$, and automatic selection) and five possible attenuating drift attenuations (*k* ranges from 6% to 10%).

**Empirical validation approach:** Previous work evaluated the performance of modified APC models (Power-5 and Poisson models) by empirically comparing retrospective projections of cancer incidence in Nordic countries [25]. The findings of that study cannot be directly applied to other countries with different characteristics of cancer epidemiology and control, but rather indicate that an empirical validation approach should be adopted to achieve optimal models for cancer projection. In our study, we empirically validated the performance of 7020 models (90 model variants × 39 gender–site combinations × 2 data) by applying fourfold time series cross-validation to the available data (1975–2019) to generate 15-year retrospective projections from four different projection bases (1975–1989, 1975–1994, 1975–1999, and 1975–2004). We then evaluated the projection performance by estimating and comparing absolute relative bias,

(5) $$ARB=\frac{\left|Predicted-Observed\right|}{Observed}\times 100\%,$$

and then selected the optimal models on the basis of the lowest median ARB across the different projection bases and periods. The data-, gender-, and site-specific information of selected parameters are in Supplementary Table S1, with the median ARB range of 0% to 7.36%, suggesting the relatively good performance of the selected models.

**Statistical analysis:** We applied the validated models to incidence and mortality data in 1987–2019 to project cancer cases and deaths forward to 2020–2054 (Supplementary Table S1). We computed all-site and all-gender combined cancer cases/deaths by aggregating cancer cases/deaths of all cancer sites and genders, respectively. We estimated the annual average cancer cases/deaths as the arithmetic mean of each 5-year period, and then calculated the changes in cancer cases/deaths over 2020–2054 by subtracting the annual average of period 2015–2019 from 2050–2054, and dividing them by the period 2015–2019 to determine the percentage changes. We estimated the ASIR and ASMR values using the “Standard Japanese Population in 1985” to be consistent with earlier studies in Japan [7,34]. Additionally, we calculated age-specific rates for eight age groups including children (aged 0–14 years), adolescents (15–19 years), young adults (20–39 years), middle-aged subjects (40–64 years), youngest-old (65–74 years), middle-old (75–84 years), and oldest-old (85+ years). Lastly, we performed a sensitivity analysis of drift attenuation on long-term cancer projections by estimating ASIR and ASMR in 2020–2054 with different assumed drift attenuations (k% ranges from 4% to 12%).

**Decomposition analysis:** We decomposed changes in cancer cases/deaths into demographic components (i.e., population size and population aging) and epidemiological components (i.e., change in population’s risk of being diagnosed with or dying from cancer due to a combination of changes in cancer risk and improvements in diagnostic or treatment practices; hereinafter, population risk) to evaluate the impact of demographic transition on the cancer burden in Japan [14,35]. We adopted a newly validated formula that proportionately allocates the three-way interaction term to all three components of changes in population age structure, population size, and population risk. This formula is superior to the previous ones as its decomposed results are stable and do not depend on the choice of reference year. All decomposition methods and formulas were presented in detail elsewhere [36].

## 3. Results

**Figure 1:**
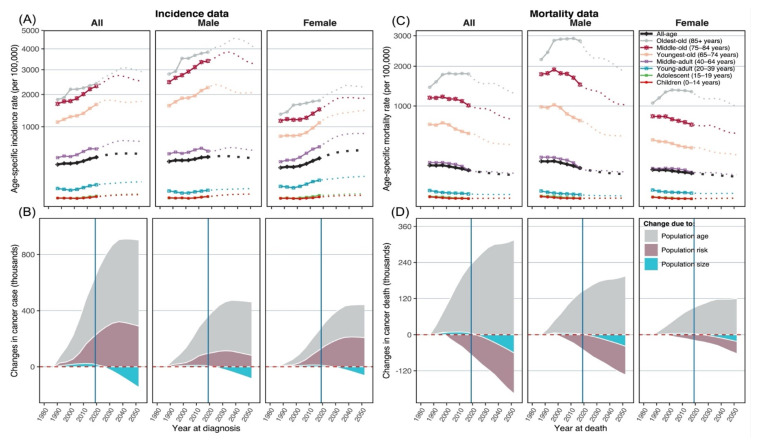
Projections of age-specific incidence rates (Panel (**A**)) and mortality rates (Panel (**C**)), and decomposed changes in new cancer cases (Panel (**B**)) and deaths (Panel (**D**)).

All-site combined incidence results, including new cancer cases, ASIR, and decomposition analysis of case changes between 2020 and 2054, are presented in Table 1. We observed an annual average of 936,570 new cancer cases (534,326 men, 402,244 women) in 2015–2019, and we projected an annual average of 1,097,567 new cases (571,065 men, 526,502 women) in 2020–2054. An increase of 36,739 cases (+6.9%) among Japanese men was completely attributable to population aging (+194,657 cases, +36.4%), while the increase in 124,258 cases (+30.9%) among Japanese women was predominantly attributable to both population aging (+101,654 cases, +25.3%) and changes in population risk (+130,407 cases, +32.4%). Over the period 2020–2054, we predicted a slightly reduced ASIR of all ages and all sites combined among men (from 374.0 to 357.3), and an increased ASIR among women (from 353.5 to 487.6). In age-specific incidence rates, we projected the leveled-off trends in most age groups among men but momentous increases in most age groups among women (Figure 1A). The proportions of older patients among total cancer cases were projected to continue increasing over 2020–2054 along with population aging, with a notable increase in oldest-old cancer patients from 14.0% to 25.9% (Supplementary Table S2). Figure 1B illustrates the trends and projection of decomposed changes in all-site combined cases over 1985–2054 (the period 1985–1989 was used as the reference). In both genders, epidemiological (population risk) and demographic (population aging) components contributed to increased cancer cases in 1985–2019, but population size (population decline) contributed to reduced cancer cases in 2020–2054. Trends in age-specific incidence rates and decomposed case changes in all 22 cancer sites are in Supplementary Figures S2–S23, with population risk contributing to the increased cases in 2020–2054 in most cancer sites, except stomach, liver, cervix uteri, gallbladder, and larynx.

Notes: Log scales were applied to the Y-axis; in panels (**A, C**), solid lines describe the observed rates, and dotted lines describe the projected rates; in panels (**B, D**), period 1980–1985 was used as a reference.

**Figure 2:**
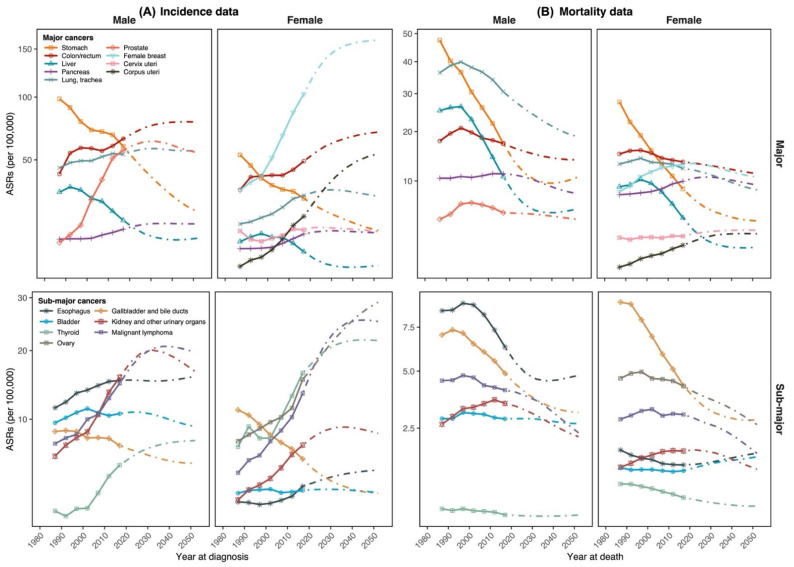
Projections of age-standardized incidence and mortality rates for major and sub-major cancer sites by gender, 1985–2054. (**A**) Incidence data and (**B**) Mortality data.

The site-specific incidence results, including new cancer cases, ASIR, rank of incidence burden, and decomposition analysis of case changes, of all major and sub-major cancer sites by gender are shown in Table 2 (other cancer sites are in Supplementary Table S3). Over the period 2020–2054, the majority of cancer sites showed increased new cases in both genders, predominantly attributed to population aging and population risk. Contrarily, some cancer sites had decreased cases including stomach (−53.3% among men, −36.5% among women), liver (−42.0%, −45.9%), and gallbladder (−8.0%, −42.9%), principally due to reduction in population risk. Specifically, decreasing ASIR was observed in cancers of the stomach, liver, and gallbladder in both genders. Demonstration of trends in and projections of site-specific ASIR by gender for major and sub-major cancer sites are shown in Figure 2A (ASIR of other cancer sites are in Supplementary Figure S24, and site-specific trends of cases are shown in Figure S25). The major cancer sites with a substantial increase in ASIR were colon/rectum (both men and women), prostate (men), and breast (women), while those in sub-major sites were malignant lymphoma (both genders), kidney (men), thyroid, and ovary (women). Our sensitivity analysis showed the modest impact of assumed drift attenuations (k% ranges from 4% to 12%) on ASIR in 2020–2054 in most cancer sites, except for male colon/rectum and female breast cancers (Supplementary Figure S26). The top three cancer incidence burdens by age group are in Table 3 (all-gender combined) and Supplementary Table S4 (by male and female). Generally, prostate and female breast cancers will be the highest incidence burdens in 2020–2054 among Japanese men and women (ahead of colon/rectum and lung cancers).

Notes: ASRs = age-standardized rates; log scales were applied to the *Y*-axis; trends in and projections of age-standardized incidence and mortality rates for other cancer sites are in Supplementary Figure S24.

Table 1 also presents all-site combined mortality results, with an annual average of 355,070 deaths (210,654 men, 144,416 women) observed in 2015–2019, decreasing to 307,870 deaths (175,797 men, 132,043 women) projected in 2020–2054. While population aging significantly added cancer deaths to both genders (+45.9% among men, +40.7% among women), the observed changes in overall deaths (−16.5%, −8.6%) were attributed to population decline (−23.5%, −22.7%) and population risk (−38.9%, −26.5%) among men and women, respectively. In the all-age and all-site combined age-standardized mortality rate (ASMR), there were substantial decreases in mortality rates among both men (from 125.5 to 85.1) and women (from 90.0 to 70.3). In age-specific mortality rates, we projected considerable reductions in mortality rates in most age groups in both genders over 2020–2054 (Figure 1C). In Figure 1D, population aging contributed to expanded cancer deaths across 1985–2054, while population risk and population decline jointly supported the diminished deaths in most of the study period. Similar visualizations of age-specific mortality rates and decomposed death changes of all 22 cancer sites are in Supplementary Figures S2–S23, with population risk contributing to the increased deaths in 2020–2054 in some cancer sites, including female breast, female pancreas, prostate, corpus uteri, bladder, and kidney cancers. Age-specific cancer deaths and proportion to all-age combined deaths are in Supplementary Table S2.

Table 4 presents cancer deaths, ASMR, ranks of mortality burden, and decomposition analysis of death changes by gender of all major and sub-major cancer sites (other cancer sites are in Supplementary Table S5). The majority of cancer sites showed reductions in deaths over the period 2020–2054 due to the decreased influence of population risk, while some had raised cancer deaths due to enormous contributions from population aging (e.g., bladder in both genders, prostate among men, colon/rectum, and pancreas among women). Visualizations of trends and projections of site-specific ASMR for major and sub-major cancer sites are in Figure 2B (ASMR of other cancer sites in Supplementary Figure S24; site-specific trends of deaths in Figure S27. The declining ASMR was observed in most cancer sites and both genders over 2020–2054, except cervix uteri (increased from 2.9 to 3.4), corpus uteri (from 2.1 to 3.1), and esophagus (1.3 to 1.7). Supplementary Figure S26 shows the minor impact of attenuation assumption on the projected ASMR in 2020–2054 in most cancer sites, except stomach and liver (both genders), male lung, and female breast cancers. While pancreas cancer will replace stomach cancer to be one of the three highest mortality burdens among the Japanese population in 2020–2054 (Table 3), the top three cancer mortality burdens will remain the same among men (lung, colon/rectum, and stomach cancers) and women (lung, colon/rectum, and pancreas cancers) across 2020–2054 (Supplementary Table S4).

## 4. Discussion

We applied empirically validated statistical models to provide long-term projections of cancer incidence and mortality burden in Japan up to 2054. For cancer incidence, we projected a slight reduction in all-site combined ASIR among men over 2020–2054, which is comparable to recent studies in the US [18], the UK [20], Australia [22], and Switzerland [23]. In contrast, the projected substantial increase in all-site combined ASIR among women is unique to Japan, probably contributed to by the observed increasing female ASIR of some cancer sites (i.e., colorectal, ovary, and esophagus) in Japan versus decreasing or stabilizing rates in other developed countries. For cancer mortality, similar to global projections [2], we predicted reductions in all-site combined and most site-specific ASMR in both genders in Japan, which is possibly related to recent reductions in cancer risk factors (e.g., tobacco smoking, infections), improvements in cancer diagnosis, treatment, and disease management, as well as reported increases in cancer survival rates [37,38]. Despite the divergent trends in ASIR between genders, both genders showed increases in cancer cases (+6.9% among men, +30.9% among women), which were predominantly a consequence of population aging (among men) or a combination of population aging and population risk (among women). Furthermore, the overall number of cancer deaths was projected to decrease among both men (−16.5%) and women (−8.6%) due to the reduction effects of population decline and population risk. While colorectal and lung cancers were predicted to remain the leading causes of cancer incidence and mortality over 2020–2054 in both genders, female breast and prostate cancers were predicted to have the highest incidence burdens in 2020–2054 among Japanese women and men, respectively.

Our findings highlight the continuous presence of colorectal and lung cancers as the largest burdens in both genders (Table 3). Although the majority of lung cancer cases are caused by tobacco smoking (67.5% in Japan) [39], the attributable fractions and magnitudes of association of smoking behavior to incidence rates are divergent between histological types [40]. Specifically, the considerable reduction in smoking prevalence from 1965 to 2018 among both Japanese men (from 82.3% to 27.8%) and women (from 15.5% to 8.7%) might have contributed to the reported declines in lung squamous non-small-cell carcinoma and small-cell carcinoma [34,41]. However, our projected leveling-off trends of lung cancer ASIR in both genders could be explained by the substantial increases in localized and distant lung adenocarcinoma, which might be connected to the recent expansion and utilization of improved diagnostic and screening techniques [34]. In contrast, the increasing incidence rates of colorectal cancer in both genders could be explained by the spread of Westernized lifestyles [42] or the introduction of organized screening programs (fecal occult blood test) [43]. The Japanese government provides screening guidelines based on efficacy evaluation and recommends annual participation in population-based and/or workplace-based screening programs for lung, colorectal, breast, cervical, and gastric cancers. While participation rates in lung cancer screening are reported to have increased among both genders, those in colorectal cancer screening are relatively low for both fecal occult blood tests (47.8% among men, 40.9% among women) and follow-up examinations (69.8% in both genders) [44]. The current slow progress in achieving national targets for colorectal cancer screening (50% in fecal occult blood tests and 90% in follow-up examinations) may not secure further reductions in colorectal mortality rate shown in a microsimulation study [45]. Nevertheless, our decomposition analysis showed the large contribution of population risk to the increases in lung and colorectal cancer cases over 2020–2054 (Supplementary Figures S3 and S6), which were shown to be possibly reducible by effective cancer control measures, e.g., controlling risk factors (tobacco smoking and lifestyles) and improving diagnosis (cancer screening) [7]. Our findings warrant greater effort from the government and policymakers to promote primary and secondary preventive interventions aimed at reducing the incidence and mortality of lung and colorectal cancers in Japan.

Female breast and prostate cancers were predicted to be the highest incidence burdens in 2020–2054 among Japanese women and men, respectively. The increasing ASIR of female breast cancer was likely contributed to by the changing reproductive factors among Japanese women (i.e., lower parity, older age at first parturition, or earlier menarche) [46], which also contributed to the increasing ASIR of other cancers that share common reproductive risk factors (i.e., ovary and corpus uteri). Another potential contributor was the increase in breast cancer screening rate (mammography) among Japanese women [44], which might be reflected in the reported increases in early-stage cancer and CIS of the breast [47]. Notably, female breast cancer will account for 25.1% and 12.1% of total new cancer cases in 2020–2054 among women and both genders combined, respectively (Supplementary Table S4 and Table 3), indicating the massive economic burden of treatment costs for breast cancer patients in the foreseeable future. Similarly, prostate cancer diagnosed by prostate-specific antigen (PSA) screening is projected to be the top burden in male cancer incidence in 2020–2054, accounting for one-fifth of new cases among men (Supplementary Table S4). The threefold increase in PSA screening in 2003 (widely adopted as an organized screening program in many municipalities) was subsequently reflected in the increasing localized prostate rate and decreasing distant prostate cancer rate in Japan after 2004, which included the possibility of overdiagnosis [48,49]. Generally, thyroid, prostate, and female breast cancers were considered to be subject to potential overdiagnosis in Japan, sharing the common factors of (1) wide adoption of minimally invasive tests (e.g., ultrasonography for thyroid, PSA for prostate, mammography for breast), and (2) a rapid increase in incidence without a corresponding change in mortality [50,51,52]. Our decomposition findings show that the majority of increasing cases of prostate and female breast cancers over 2020–2054 are attributable to population risk (Supplementary Figures S7 and S8), suggesting room for improving preventive interventions in the overdiagnosis context. Therefore, further studies using statistical or mathematical modeling approaches are needed to avoid overdiagnosis [53].

Among all cancer sites, only cancers of the stomach, liver, and gallbladder showed a substantial decrease in incidence and mortality (regarding both number of cancer cases/deaths and ASR) in both genders over 2020–2054. Our decomposition analysis showed the large contribution of population risk to the decreased number of cases of those infection-associated cancers over 2020–2054 (Supplementary Figures S2 and S4), emphasizing the necessity of controlling oncogenic infections. For example, Japan has included the eradication of *Helicobacter pylori* (*H. pylori*) in its universal health insurance for the treatment of chronic gastritis, gastric, and duodenal ulcers since 2013 [54]. Thus, the decreasing incidence of stomach cancer is mainly attributed to the tremendous decrease in *H. pylori* infection, in combination with improvements in diagnosis, diet, and food preservation techniques in Japan [55]. Similarly, reduction in risk factors for liver and gallbladder cancers (i.e., hepatitis B and C viruses, chronic infections, and obesity) was the predominant contributing factor to the decreasing incidence rates of these cancers among the Japanese population [56]. Our findings emphasize the important role of policymakers and governments in reforming cancer policies for controlling cancer risk factors, including oncogenic infections. However, the growing incidence of esophageal adenocarcinoma is believed to be related to the reduction in *H. pylori* infection, which plays a protective role in the presence of gastroesophageal reflux disease and Barrett’s esophagus [57,58]. Thus, further investigations are required to summarize lessons learned for other low- and middle-income countries regarding health policies toward universal health coverage (e.g., improving communicable and noncommunicable disease management) to achieve better cancer control outcomes [59,60].

Notably, we projected neither age-standardized incidence nor mortality rates of cervical cancer significantly decreasing over 2020–2054, and only a modest contribution of population risk to the reduction of this infection-associated cancer burden in Japan (Supplementary Figure S9). As cervical cancer can be effectively prevented by nationwide organized screening [61], in combination with human papillomavirus (HPV) vaccination programs [62], our results suggest the currently modest effectiveness of cervical cancer control programs in Japan. Organized screening coverage in Japan is lower than in other developed countries (i.e., 43.7% in Japan compared with 51.9% in Korea, 72.6% in the US, and 74.4% in the UK), although Japan introduced (since the 1950s) and integrated (since 1982) cytology screening programs into its national system early on [63,64]. Additionally, Japan’s HPV vaccination coverage is poor (<1%) [65], despite the fact that the Japanese government has recently confirmed the safety of this vaccination by ending the restriction on active recommendation and strengthening the support system for HPV vaccination [66]. Hence, our findings warrant greater efforts in improving the effectiveness of cervical cancer control programs in Japan, particularly the cervical cancer screening rate and HPV vaccination coverage, to better control cervical cancer burdens in the future [67].

We applied decomposition analysis of changes in cancer cases and deaths over 2020–2054 to further assess the burden of population aging on cancer incidence and mortality in Japan. Given the rapid escalation in the proportion of older people (≥65 years), we show that population aging is the major contributing factor to the increase in cancer cases (36.5% among men, 25.3% among women) and deaths (45.8% among men, 40.5% among women) over the study period (Table 1). We projected a dramatic escalation in the number of oldest-old cancer patients, from 14.0% in 2015–2019 to 25.9% in 2020–2054 (Supplementary Table S2). These older patients generally experience a combination of comorbidities, frailty, functional limitations, and other age-associated conditions, in addition to cancer. This situation, therefore, requires the development of tailored and supportive interventions which not only aim to reduce mortality but also to improve the quality of life for these elders [68]. Although the “Clinical practice guidelines of cancer drug therapies for the elderly”, published in 2019, needs further improvement (e.g., the inclusion of more common carcinomas, higher levels of evidence, and more discussion on supportive and symptomatic therapies), it is the first and most qualified reference for the concept of elderly cancer pharmacotherapy in Japan [69]. Additionally, the ongoing reforms of social security systems in Japan (e.g., medical care, nursing care, and pensions) are expected to adapt to the complex challenges of population aging and decline, including the shortage of medical and human resources, and the decreasing sustainability of the finance and healthcare system [70]. Our projections of cancer burden will serve as qualified inputs and warrant further health economics and policy research in investigating future costs of cancer diagnosis and treatment, securing medical and human resources, and establishing evidence-based control programs [71].

This study provides the first comprehensive projections of cancer incidence and mortality for 22 cancer sites in both genders in Japan, using empirically validated APC models applied to the most up-to-date and nationally representative data. Additionally, it presents estimations and trends of decomposed changes in cancer cases and deaths over the period 2020–2054 using a newly proposed formula, which addresses limitations in the previous decomposition studies [36]. Nevertheless, several limitations of this study should be mentioned. First, we generated projections of cancer incidence/mortality on the basis of the projected population, which were forecast on the basis of prior assumptions of future migration, birth, and death rates, which in turn impact our cancer projections. Second, our APC models applied the widely accepted assumption that the last trends remained constant without substantial changes in policy nor significant improvements in human cancer control, such as new screening methods, novel vaccines, and advances in medical techniques. Thus, the presented projections may not reflect any newly implemented/future interventions/policies [72,73]; nevertheless, they still provide baseline projections against which the impact of future cancer interventions/policies or factors that appear after the study periods can be assessed (e.g., COVID-19-related delays in cancer screening and treatment) [22]. Nevertheless, a regular projection system in Japan with annual or biennial updates is recommended, as it could integrate the latest trends and provide the most stable and reliable cancer statistics for effective evidence-based cancer control. Furthermore, we did not incorporate covariates for projections of some specific cancers with potential overdiagnosis (e.g., PSA screening rate for prostate cancer and breast cancer screening rates for female breast cancer) as doing so would require further assumptions for projecting these covariates into the future, thereby risking more uncertainty. Nevertheless, future studies with different modeling approaches (e.g., microsimulation modeling studies) and incorporating explanatory variables to predict cancers with potential overdiagnosis might be useful complements and comparisons to this work.

## 5. Conclusions

In conclusion, we project cancer incidence and mortality for 22 cancer sites and both genders in Japan, which may serve as baseline projections against which to assess the impact of future cancer interventions/policies, and qualified inputs for research into health economics investigating future costs of cancer diagnosis and treatment. The decomposition analyses show a relatively large cancer burden attributed to population aging in the future, requiring the development of tailored and supportive interventions for reducing mortality and improving the quality of life for the elders. We show the decreasing incidence and mortality for infection-associated cancers and emphasize the important role of policymakers and governments in reforming cancer policies aimed at controlling cancer risk factors, including oncogenic infections. Nevertheless, we project the continued presence and rapid increase in the burden of some cancers associated with modifiable risk factors in Japan. These findings warrant greater efforts in cancer control programs, specifically in enhancing cancer screening and controlling cancer risk factors, toward a vision for a healthier Japan [74].

## Figures and Tables

**Table 1:** All-site and all-age combined cancer incidence and mortality by gender and decomposition of changes in new cancer case/death over 2020–2054.

Data	Gender	Period 2015–2019 ^a^		Period 2020–2054 ^a^		Case/Death Changes over Period 2020–2054			
		Case/Death	Rate ^b^	Case/Death	Rate ^b^	Total (%)	Population Risk (%) ^c^	Population Age Structure (%) ^d^	Population Size (%) ^e^
Incidence	All-gender	936,570	369.8	1,097,567	427.0	+160,997 (+17.2%)	+108,160 (+11.5%)	+296,311 (+31.6%)	−243,474 (−26.0%)
	Male	534,326	374.0	571,065	357.3	+36,739 (+6.9%)	−22,247 (−4.2%)	+194,657 (+36.4%)	−135,671 (−25.4%)
	Female	402,244	353.5	526,502	487.6	+124,258 (+30.9%)	+130,407 (+32.4%)	+101,654 (+25.3%)	−107,803 (−26.8%)
Mortality	All-gender	355,070	109.9	307,840	79.9	−47,230 (−13.3%)	−120,244 (−33.9%)	+155,337 (+43.7%)	−82,323 (−23.2%)
	Male	210,654	125.5	175,797	85.1	−34,857 (−16.5%)	−81,941 (−38.9%)	+96,597 (+45.9%)	−49,513 (−23.5%)
	Female	144,416	90.0	132,043	70.3	−12,373 (−8.6%)	−38,303 (−26.5%)	+58,740 (+40.7%)	−32,810 (−22.7%)

^a^ Estimated as annual average of 5-year periods; ^b^ age-standardized rate; ^c^ changes due to epidemiological factors (i.e., changes in population risks of being diagnosed with or dying from cancers, and improvements in diagnostic or treatment practices); ^d^ changes due to population age structure; ^e^ changes due to population size.

**Table 2:** Site-specific cancer incidence by gender and decomposition of case changes 2020–2054 for major and sub-major cancers.

Gender	Cancer Group	Cancer Site	ICD-10	Period 2015–2019 ^a^			Period 2050–2054 ^a^			Case Changes over Period 2020–2054			
				Case	Rate ^b^	Rank	Case	Rate ^b^	Rank	Total (%)	Population Risk (%) ^c^	Population Age Structure (%) ^d^	Population Size (%) ^e^
Male and female combined	Major	Stomach	C16	128,667	44.9	2	66,818	18.6	5	−61,849 (−48.1%)	−75,283 (−58.5%)	+36,870 (+28.7%)	−23,436 (−18.2%)
		Colon/rectum	C18–C20	151,904	58.0	1	207,435	75.1	1	+55,531 (+36.6%)	+45,279 (+29.8%)	+53,280 (+35.1%)	−43,028 (−28.3%)
		Liver	C22	39,532	13.3	7	22,438	8.4	14	−17,094 (−43.2%)	−20,969 (−53.0%)	+11,459 (+29.0%)	−7584 (−19.2%)
		Pancreas	C25	41,065	14.0	6	49,795	15.4	7	+8730 (+21.3%)	+3306 (+8.1%)	+16,287 (+39.7%)	−10,864 (−26.5%)
		Lung, trachea	C33–C34	123,105	42.5	3	146,149	44.5	2	+23,044 (+18.7%)	+7506 (+6.1%)	+47,977 (+39.0%)	−32,439 (−26.4%)
		Prostate	C61	89,466	30.3	5	114,306	30.6	4	+24,840 (+27.8%)	+10,420 (+11.6%)	+39,223 (+43.8%)	−24,804 (−27.7%)
		Breast (female)	C50	92,901	50.2	4	132,332	76.9	3	+39,431 (+42.4%)	+55,750 (+60.0%)	+9755 (+10.5%)	−26,073 (−28.1%)
		Cervix uteri	C53	10,986	7.1	19	10,036	6.6	20	−950 (−8.6%)	+1271 (+11.6%)	+173 (+1.6%)	−2394 (−21.8%)
		Corpus uteri	C54	16,581	9.6	16	38,482	25.9	9	+21,901 (+132.1%)	+27,799 (+167.7%)	+697 (+4.2%)	−6596 (−39.8%)
	Sub-major	Esophagus	C15	25,354	9.8	10	27,802	10.9	12	+2448 (+9.7%)	+1869 (+7.4%)	+7017 (+27.7%)	−6439 (−25.4%)
		Gallbladder and bile ducts	C23–C24	22,426	6.5	13	16,975	4.4	17	−5451 (−24.3%)	−9993 (−44.6%)	+9356 (+41.7%)	−4815 (−21.5%)
		Bladder	C67	22,743	7.2	12	25,737	6.5	13	+2994 (+13.2%)	−1995 (−8.8%)	+10,881 (+47.8%)	−5891 (−25.9%)
		Kidney and other urinary organs	C64–C66 C68	29,040	11.7	9	38,998	13.0	8	+9958 (+34.3%)	+7535 (+25.9%)	+10,561 (+36.4%)	−8138 (−28.0%)
		Thyroid	C73	17,954	10.7	15	22,408	14.4	15	+4454 (+24.8%)	+7834 (+43.6%)	+1362 (+7.6%)	−4742 (−26.4%)
		Malignant lymphoma	C81–C85 C96	34,265	14.2	8	57,875	22.5	6	+23,610 (+68.9%)	+21,577 (+63.0%)	+13,031 (+38.0%)	−10,998 (−32.1%)
		Ovary	C56	12,722	7.5	18	20,140	14.0	16	+7418 (+58.3%)	+10,682 (+84.0%)	+606 (+4.8%)	−3869 (−30.4%)
Male	Major	Stomach	C16	88,574	59.3	2	41,361	21.5	4	−47,213 (−53.3%)	−56,588 (−63.9%)	+25,272 (+28.5%)	−15,896 (−17.9%)
		Colon/rectum	C18–C20	86,247	64.9	3	109,737	78.0	2	+23,490 (+27.2%)	+17,888 (+20.7%)	+29,662 (+34.4%)	−24,060 (−27.9%)
		Liver	C22	26,664	18.0	5	15,471	11.6	11	−11193 (−42.0%)	−13,498 (−50.6%)	+7546 (+28.3%)	−5241 (−19.7%)
		Pancreas	C25	21,126	14.7	6	26,226	16.7	5	+5100 (+24.1%)	+2577 (+12.2%)	+8347 (+39.5%)	−5825 (−27.6%)
		Lung, trachea	C33–C34	82,290	53.6	4	97,615	56.4	3	+15,325 (+18.6%)	+3672 (+4.5%)	+33,836 (+41.1%)	−22,183 (−27.0%)
		Prostate	C61	89466	56.5	1	114,306	54.8	1	+24,840 (+27.8%)	+10,420 (+11.6%)	+39,223 (+43.8%)	−24,804 (−27.7%)
	Sub-major	Esophagus	C15	20,990	15.2	7	21,288	15.8	8	+298 (+1.4%)	−186 (−0.9%)	+5669 (+27.0%)	−5186 (−24.7%)
		Gallbladder and bile ducts	C23–C24	11,922	7.1	13	10,972	5.4	13	−950 (−8.0%)	−3568 (−29.9%)	+5472 (+45.9%)	−2854 (−23.9%)
		Bladder	C67	17,139	10.7	10	19,006	9.1	10	+1867 (+10.9%)	−1879 (−11.0%)	+8202 (+47.9%)	−4456 (−26.0%)
		Kidney and other urinary organs	C64–C66 C68	19,722	15.7	8	26,218	16.6	6	+6496 (+32.9%)	+4871 (+24.7%)	+7218 (+36.6%)	−5593 (−28.4%)
		Thyroid	C73	4663	5.2	16	6571	7.6	15	+1908 (+40.9%)	+2573 (+55.2%)	+716 (+15.4%)	−1380 (−29.6%)
		Malignant lymphoma	C81–C85 C96	18,331	14.8	9	25,718	19.7	7	+7387 (+40.3%)	+5831 (+31.8%)	+6972 (+38.0%)	−5415 (−29.5%)
Female	Major	Stomach	C16	40,093	27.8	4	25,457	14.3	6	−14,636 (−36.5%)	−18,694 (−46.6%)	+11,598 (+28.9%)	−7540 (−18.8%)
		Colon/rectum	C18–C20	65,657	49.1	2	97,698	70.0	2	+32,041 (+48.8%)	+27,391 (+41.7%)	+23,618 (+36.0%)	−18,968 (−28.9%)
		Liver	C22	12,867	7.7	9	6967	4.6	14	−5900 (−45.9%)	−7470 (−58.1%)	+3913 (+30.4%)	−2342 (−18.2%)
		Pancreas	C25	19,938	13.0	5	23,569	13.4	7	+3631 (+18.2%)	+729 (+3.7%)	+7940 (+39.8%)	−5038 (−25.3%)
		Lung, trachea	C33–C34	40,814	29.2	3	48,533	28.8	3	+7719 (+18.9%)	+3834 (+9.4%)	+14,141 (+34.6%)	−10,256 (−25.1%)
		Breast (female)	C50	92,901	103.0	1	132,332	160.2	1	+39,431 (+42.4%)	+55,750 (+60.0%)	+9755 (+10.5%)	−26,073 (−28.1%)
		Cervix uteri	C53	10,986	14.5	12	10,036	13.8	13	−950 (−8.6%)	+1271 (+11.6%)	+173 (+1.6%)	−2394 (−21.8%)
		Corpus uteri	C54	16,581	19.7	6	38,482	54.2	4	+21,901 (+132.1%)	+27,799 (+167.7%)	+697 (+4.2%)	−6596 (−39.8%)
	Sub-major	Esophagus	C15	4364	3.5	18	6513	4.8	17	+2149 (+49.2%)	+2055 (+47.1%)	+1348 (+30.9%)	−1253 (−28.7%)
		Gallbladder and bile ducts	C23–C24	10,504	5.8	13	6003	3.1	18	−4501 (−42.9%)	−6425 (−61.2%)	+3884 (+37.0%)	−1960 (−18.7%)
		Bladder	C67	5604	3.2	17	6732	3.1	16	+1128 (+20.1%)	−116 (−2.1%)	+2679 (+47.8%)	−1435 (−25.6%)
		Kidney and other urinary organs	C64–C66 C68	9318	7.1	14	12,780	8.4	11	+3462 (+37.2%)	+2664 (+28.6%)	+3343 (+35.9%)	−2545 (−27.3%)
		Thyroid	C73	13,291	16.3	8	15,837	21.7	10	+2546 (+19.2%)	+5261 (+39.6%)	+646 (+4.9%)	−3362 (−25.3%)
		Malignant lymphoma	C81–C85 C96	15,934	13.4	7	32,157	25.2	5	+16,223 (+101.8%)	+15,746 (+98.8%)	+6060 (+38.0%)	−5582 (−35.0%)
		Ovary	C56	12,722	15.4	10	20,140	29.1	8	+7418 (+58.3%)	+10,682 (+84.0%)	+606 (+4.8%)	−3869 (−30.4%)

^a^ Estimated from 5-year periods; ^b^ age-standardized rate; ^c^ changes due to epidemiological factors (i.e., changes in population risks of being diagnosed with or dying from cancers, and improvements in diagnostic or treatment practices); ^d^ changes due to population age structure; ^e^ changes due to population size; %: proportion to the number of cases in 2015–2019.

**Table 3:** Top three cancer diagnoses in 2015–2019 and 2050–2054 by age group (both genders).

Data	Age Group	Burden Rank	Period 2015–2019 ^a^			Period 2050–2054 ^a^		
			Cancer Site	ICD-10	Case/Death (%)	Cancer Site	ICD-10	Case/Death (%)
Incidence	All age	1	Colon/rectum	C18–C20	151,904 (16.2%)	Colon/rectum	C18–C20	207,435 (18.9%)
		2	Stomach	C16	128,667 (13.7%)	Lung, trachea	C33–C34	146,149 (13.3%)
		3	Lung, trachea	C33–C34	123,105 (13.1%)	Female breast	C50	132,332 (12.1%)
	Oldest-old (85+ years)	1	Colon/rectum	C18–C20	22,521 (17.2%)	Colon/rectum	C18–C20	55,172 (19.4%)
		2	Stomach	C16	19,684 (15.0%)	Lung, trachea	C33–C34	41,747 (14.7%)
		3	Lung, trachea	C33–C34	19,300 (14.7%)	Prostate	C61	32,077 (11.3%)
	Middle-old (75–84 years)	1	Colon/rectum	C18–C20	44,914 (16.2%)	Colon/rectum	C18–C20	69,307 (18.9%)
		2	Stomach	C16	44,599 (16.1%)	Lung, trachea	C33–C34	55,801 (15.2%)
		3	Lung, trachea	C33–C34	41,414 (14.9%)	Prostate	C61	48,743 (13.3%)
	Youngest-old (65–74 years)	1	Colon/rectum	C18–C20	48,260 (16.6%)	Colon/rectum	C18–C20	47,693 (20.1%)
		2	Lung, trachea	C33–C34	42,430 (14.6%)	Lung, trachea	C33–C34	32,848 (13.8%)
		3	Stomach	C16	42,389 (14.6%)	Prostate	C61	27,218 (11.5%)
	Middle-adult (40–64 years)	1	Female breast	C50	45,392 (20.9%)	Female breast	C50	45,448 (23.7%)
		2	Colon/rectum	C18–C20	34,526 (15.9%)	Colon/rectum	C18–C20	33,667 (17.6%)
		3	Stomach	C16	21,156 (9.7%)	Corpus uteri	C54	18,232 (9.5%)
	Young-adult (20–39 years)	1	Female breast	C50	3967 (22.1%)	Female breast	C50	5047 (31.7%)
		2	Thyroid	C73	2390 (13.3%)	Thyroid	C73	1627 (10.2%)
		3	Cervix uteri	C53	1915 (10.7%)	Colon/rectum	C18–C20	1544 (9.7%)
	Adolescent (15–19 years)	1	Leukemia	C91–C95	180 (25.0%)	Malignant lymphoma	C81–C85 C96	202 (27.3%)
		2	Malignant lymphoma	C81–C85 C96	121 (16.8%)	Leukemia	C91–C95	179 (24.2%)
		3	Thyroid	C73	116 (16.1%)	Brain, nervous system	C70–C72	87 (11.8%)
	Children (0–14 years)	1	Leukemia	C91–C95	744 (46.8%)	Leukemia	C91–C95	681 (40.0%)
		2	Brain, nervous system	C70–C72	327 (20.6%)	Malignant lymphoma	C81–C85 C96	492 (28.9%)
		3	Malignant lymphoma	C81–C85 C96	237 (14.9%)	Brain, nervous system	C70–C72	334 (19.6%)
Mortality	All age	1	Lung, trachea	C33–C34	74,407 (21.0%)	Lung, trachea	C33–C34	61,980 (20.1%)
		2	Colon/rectum	C18–C20	50,508 (14.2%)	Colon/rectum	C18–C20	50,737 (16.5%)
		3	Stomach	C16	44,910 (12.6%)	Pancreas	C25	34,741 (11.3%)
	Oldest-old (85+ years)	1	Lung, trachea	C33–C34	19,165 (19.8%)	Lung, trachea	C33–C34	24,317 (19.5%)
		2	Colon/rectum	C18–C20	14,892 (15.4%)	Colon/rectum	C18–C20	22,074 (17.7%)
		3	Stomach	C16	12,994 (13.4%)	Pancreas	C25	13,963 (11.2%)
	Middle-old (75–84 years)	1	Lung, trachea	C33–C34	26,988 (22.3%)	Lung, trachea	C33–C34	24675 (22.7%)
		2	Colon/rectum	C18–C20	15,797 (13.0%)	Colon/rectum	C18–C20	16,496 (15.2%)
		3	Stomach	C16	15,483 (12.8%)	Pancreas	C25	12,802 (11.8%)
	Youngest-old (65–74 years)	1	Lung, trachea	C33–C34	20,575 (23.1%)	Lung, trachea	C33–C34	9582 (20.3%)
		2	Colon/rectum	C18–C20	12,410 (13.9%)	Colon/rectum	C18–C20	7658 (16.2%)
		3	Stomach	C16	11,120 (12.5%)	Pancreas	C25	5095 (10.8%)
	Middle-adult (40–64 years)	1	Lung, trachea	C33–C34	7558 (16.4%)	Colon/rectum	C18–C20	4365 (16.9%)
		2	Colon/rectum	C18–C20	7146 (15.5%)	Lung, trachea	C33–C34	3348 (12.9%)
		3	Stomach	C16	5076 (11.0%)	Stomach	C16	2844 (11.0%)
	Young-adult (20–39 years)	1	Female breast	C50	268 (14.0%)	Female breast	C50	225 (21.3%)
		2	Colon/rectum	C18–C20	257 (13.4%)	Brain, nervous system	C70–C72	149 (14.1%)
		3	Stomach	C16	234 (12.2%)	Colon/rectum	C18–C20	141 (13.4%)
	Adolescent (15–19 years)	1	Leukemia	C91–C95	34 (44.2%)	Brain, nervous system	C70–C72	28 (45.9%)
		2	Brain, nervous system	C70–C72	21 (27.3%)	Leukemia	C91–C95	17 (27.9%)
		3	Malignant lymphoma	C81–C85 C96	7 (9.1%)	Malignant lymphoma	C81–C85 C96	6 (9.8%)
	Children (0–14 years)	1	Brain, nervous system	C70–C72	88 (45.1%)	Brain, nervous system	C70–C72	98 (58.7%)
		2	Leukemia	C91–C95	78 (40.0%)	Leukemia	C91–C95	48 (28.7%)
		3	Liver	C22	9 (4.6%)	Malignant lymphoma	C81–C85 C96	7 (4.2%)

^a^ Estimated as annual average of 5-year periods.

**Table 4:** Site-specific cancer mortality by gender and decomposition of death changes in 2020–2054 for major and sub-major cancers.

Gender	Cancer Group	Cancer Site	ICD-10	Period 2015–2019 ^a^			Period 2050–2054 ^a^			Death Changes over Period 2020–2054			
				Death	Rate ^b^	Rank	Death	Rate ^b^	Rank	Total (%)	Population Risk (%) ^c^	Population Age Structure (%) ^d^	Population Size (%) ^e^
Male and female combined	Major	Stomach	C16	44,910	13.4	3	29,123	7.9	4	−15,787 (−35.2%)	−24,870 (−55.4%)	+18,435 (+41.0%)	−9352 (−20.8%)
		Colon/rectum	C18–C20	50,508	15.6	2	50,737	12.9	2	+229 (+0.5%)	−10,571 (−20.9%)	+23,275 (+46.1%)	−12,474 (−24.7%)
		Liver	C22	27,143	8.0	5	13,014	4.1	7	−14,129 (−52.1%)	−18,662 (−68.8%)	+9649 (+35.5%)	−5116 (−18.8%)
		Pancreas	C25	34,261	10.7	4	34,741	8.7	3	+480 (+1.4%)	−5925 (−17.3%)	+14,812 (+43.2%)	−8408 (−24.5%)
		Lung, trachea	C33–C34	74,407	22.1	1	61,980	14.4	1	−12427 (−16.7%)	−28,935 (−38.9%)	+33,581 (+45.1%)	−17,074 (−22.9%)
		Prostate	C61	11,987	2.8	9	15,546	2.6	5	+3559 (+29.7%)	−2171 (−18.1%)	+9292 (+77.5%)	−3561 (−29.7%)
		Breast (female)	C50	14,275	6.3	7	12,804	5.1	8	−1471 (−10.3%)	−1617 (−11.3%)	+3276 (+22.9%)	−3130 (−21.9%)
		Cervix uteri	C53	2822	1.4	17	3287	1.6	18	+465 (+16.5%)	+649 (+23.0%)	+534 (+18.9%)	−717 (−25.4%)
		Corpus uteri	C54	2487	1.0	19	3791	1.5	16	+1304 (+52.4%)	+1296 (+52.1%)	+747 (+30.0%)	−738 (−29.7%)
	Sub-major	Esophagus	C15	11,550	4.0	10	10,193	3.4	11	−1357 (−11.7%)	−2543 (−22.0%)	+3900 (+33.8%)	−2714 (−23.5%)
		Gallbladder and bile ducts	C23–C24	18,091	4.7	6	14,621	3.0	6	−3470 (−19.2%)	−8521 (−47.1%)	+9161 (+50.6%)	−4110 (−22.7%)
		Bladder	C67	8577	2.1	13	11,550	2.2	9	+2973 (+34.7%)	−543 (−6.3%)	+6052 (+70.6%)	−2537 (−29.6%)
		Kidney and other urinary organs	C64-C66 C68	9340	2.7	11	7757	1.8	12	−1583 (−16.9%)	−3883 (−41.6%)	+4445 (+47.6%)	−2144 (−23.0%)
		Thyroid	C73	1785	0.5	20	1699	0.4	21	−86 (−4.8%)	−515 (−28.9%)	+858 (+48.1%)	−429 (−24.0%)
		Malignant lymphoma	C81–C85 C96	12,483	3.6	8	10,471	2.1	10	−2012 (−16.1%)	−5237 (−42.0%)	+6074 (+48.7%)	−2849 (−22.8%)
		Ovary	C56	4739	2.1	15	3801	1.2	15	−938 (−19.8%)	−1047 (−22.1%)	+1092 (+23.0%)	−983 (−20.7%)
Male	Major	Stomach	C16	29,457	17.3	2	19,634	10.6	3	−9823 (−33.3%)	−15,783 (−53.6%)	+12,312 (+41.8%)	−6352 (−21.6%)
		Colon/rectum	C18–C20	27135	17.3	3	26,083	13.9	2	−1052 (−3.9%)	−6420 (−23.7%)	+12,136 (+44.7%)	−6768 (−24.9%)
		Liver	C22	17,824	10.7	4	9100	5.8	6	−8724 (−48.9%)	−11,508 (−64.6%)	+6284 (+35.3%)	−3500 (−19.6%)
		Pancreas	C25	17,341	11.2	5	15,195	8.0	5	−2146 (−12.4%)	−5120 (−29.5%)	+7077 (+40.8%)	−4103 (−23.7%)
		Lung, trachea	C33–C34	52,872	30.6	1	42,325	18.8	1	−10,547 (−19.9%)	−22,530 (−42.6%)	+24,172 (+45.7%)	−12,189 (−23.1%)
		Prostate	C61	11,987	5.4	6	15,546	4.6	4	+3559 (+29.7%)	−2171 (−18.1%)	+9292 (+77.5%)	−3561 (−29.7%)
	Sub-major	Esophagus	C15	9562	6.3	7	7421	4.8	9	−2141 (−22.4%)	−3048 (−31.9%)	+3059 (+32.0%)	−2152 (−22.5%)
		Gallbladder and bile ducts	C23–C24	9199	4.9	8	7849	3.1	7	−1350 (−14.7%)	−4104 (−44.6%)	+4963 (+54.0%)	−2209 (−24.0%)
		Bladder	C67	5842	2.8	11	7457	2.7	8	+1615 (+27.6%)	−880 (−15.1%)	+4214 (+72.1%)	−1719 (−29.4%)
		Kidney and other urinary organs	C64–C66 C68	5955	3.5	10	4906	2.2	11	−1049 (−17.6%)	−2529 (−42.5%)	+2875 (+48.3%)	−1394 (−23.4%)
		Thyroid	C73	582	0.3	18	615	0.3	18	+33 (+5.7%)	−101 (−17.4%)	+288 (+49.5%)	−154 (−26.5%)
		Malignant lymphoma	C81–C85 C96	6964	4.1	9	5916	2.3	10	−1048 (−15.0%)	−2969 (−42.6%)	+3571 (+51.3%)	−1650 (−23.7%)
Female	Major	Stomach	C16	15,453	8.7	4	9488	4.4	5	−5965 (−38.6%)	−9088 (−58.8%)	+6123 (+39.6%)	−3000 (−19.4%)
		Colon/rectum	C18–C20	23,373	13.5	1	24,654	11.3	1	+1281 (+5.5%)	−4151 (−17.8%)	+11,139 (+47.7%)	−5707 (−24.4%)
		Liver	C22	9320	4.8	6	3915	2.0	9	−5405 (−58.0%)	−7154 (−76.8%)	+3365 (+36.1%)	−1616 (−17.3%)
		Pancreas	C25	16,920	10.0	3	19,545	9.3	3	+2625 (+15.5%)	−805 (−4.8%)	+7735 (+45.7%)	−4305 (−25.4%)
		Lung, trachea	C33–C34	21,535	12.2	2	19,655	8.6	2	−1880 (−8.7%)	−6405 (−29.7%)	+9410 (+43.7%)	−4885 (−22.7%)
		Breast (female)	C50	14,275	13.0	5	12,804	10.7	4	−1471 (−10.3%)	−1617 (−11.3%)	+3276 (+22.9%)	−3130 (−21.9%)
		Cervix uteri	C53	2822	2.9	12	3287	3.4	12	+465 (+16.5%)	+649 (+23.0%)	+534 (+18.9%)	−717 (−25.4%)
		Corpus uteri	C54	2487	2.1	14	3791	3.1	11	+1304 (+52.4%)	+1296 (+52.1%)	+747 (+30.0%)	−738 (−29.7%)
	Sub-major	Esophagus	C15	1988	1.3	17	2772	1.7	14	+784 (+39.4%)	+505 (+25.4%)	+841 (+42.3%)	−562 (−28.3%)
		Gallbladder and bile ducts	C23–C24	8892	4.3	7	6772	2.8	6	−2120 (−23.8%)	−4417 (−49.7%)	+4198 (+47.2%)	−1901 (−21.4%)
		Bladder	C67	2735	1.2	13	4093	1.6	8	+1358 (+49.7%)	+338 (+12.4%)	+1838 (+67.2%)	−818 (−29.9%)
		Kidney and other urinary organs	C64–C66 C68	3385	1.7	11	2851	1.2	13	−534 (−15.8%)	−1354 (−40.0%)	+1570 (+46.4%)	−750 (−22.2%)
		Thyroid	C73	1203	0.6	18	1084	0.5	19	−119 (−9.9%)	−414 (−34.4%)	+570 (+47.4%)	−275 (−22.9%)
		Malignant lymphoma	C81–C85 C96	5519	3.0	8	4555	1.7	7	−964 (−17.5%)	−2268 (−41.1%)	+2503 (+45.4%)	−1198 (−21.7%)
		Ovary	C56	4739	4.3	9	3801	2.6	10	−938 (−19.8%)	−1047 (−22.1%)	+1092 (+23.0%)	−983 (−20.7%)

^a^ Estimated as annual average of 5-year periods; ^b^ age-standardized rate; ^c^ changes due to epidemiological factors (i.e., changes in population risks of being diagnosed with or dying from cancers, improvements in diagnostic or treatment practices); ^d^ changes due to population age structure; ^e^ changes due to population size. %: proportion to the number of deaths in 2015–2019.

## Data Availability

The data that support the findings of this study are openly available from Cancer Statistics in Japan at https://ganjoho.jp/ (accessed on 16 June 2022) reference number [27,28] and the Institute of Population and Social Security at https://www.ipss.go.jp/ (accessed on 16 June 2022) reference number [29]. The R codes for statistical analysis and data visualization are available for research purposes upon reasonable request to the corresponding author.

## Supplementary Materials

The following supporting information can be downloaded at https://www.mdpi.com/article/10.3390/cancers14246076/s1., Table S1: Information of empirically selected models with data-, gender- and site-specific parameters; Table S2: Observed and projected new cancer cases and deaths in 2015–2019 and 2050–2054 by age groups and genders; Table S3: Cancer incidence by genders and decomposed changes in cases over 2020–2054 for other cancers; Table S4: Observed and projected top burden of cancer in Japan by genders and age groups; Table S5: Cancer mortality by genders and decomposed changes in deaths over 2020–2054 for other cancers; Figure S1: Trend in and projection of total population, proportions of people aged 65+ and 85+, and population pyramids in Japan, 1985–2055 10; Figure S2: Projections of age-specific incidence and mortality rates and decomposed changes in new cases and deaths — Stomach; Figure S3: Projections of age-specific incidence and mortality rates and decomposed changes in new cases and deaths — Colon/rectum; Figure S4: Projections of age-specific incidence and mortality rates and decomposed changes in new cases and deaths — Liver; Figure S5: Projections of age-specific incidence and mortality rates and decomposed changes in new cases and deaths — Pancreas; Figure S6: Projections of age-specific incidence and mortality rates and decomposed changes in new cases and deaths — Lung, trachea; Figure S7: Projections of age-specific incidence and mortality rates and decomposed changes in new cases and deaths — Prostate; Figure S8: Projections of age-specific incidence and mortality rates and decomposed changes in new cases and deaths — Female breast; Figure S9: Projections of age-specific incidence and mortality rates and decomposed changes in new cases and deaths — Cervix uteri; Figure S10: Projections of age-specific incidence and mortality rates and decomposed changes in new cases and deaths — Corpus uteri; Figure S11: Projections of age-specific incidence and mortality rates and decomposed changes in new cases and deaths — Esophagus; Figure S12: Projections of age-specific incidence and mortality rates and decomposed changes in new cases and deaths — Gallbladder and bile ducts; Figure S13: Projections of age-specific incidence and mortality rates and decomposed changes in new cases and deaths — Bladder; Figure S14: Projections of age-specific incidence and mortality rates and decomposed changes in new cases and deaths — Kidney and other urinary organs; Figure S15: Projections of age-specific incidence and mortality rates and decomposed changes in new cases and deaths — Thyroid; Figure S16: Projections of age-specific incidence and mortality rates and decomposed changes in new cases and deaths — Malignant lymphoma; Figure S17: Projections of age-specific incidence and mortality rates and decomposed changes in new cases and deaths — Ovary; Figure S18: Projections of age-specific incidence and mortality rates and decomposed changes in new cases and deaths — Oral cavity and pharynx; Figure S19: Projections of age-specific incidence and mortality rates and decomposed changes in new cases and deaths — Larynx; Figure S20: Projections of age-specific incidence and mortality rates and decomposed changes in new cases and deaths — Skin; Figure S21: Projections of age-specific incidence and mortality rates and decomposed changes in new cases and deaths — Brain, nervous system; Figure S22: Projections of age-specific incidence and mortality rates and decomposed changes in new cases and deaths — Multiple myeloma; Figure S23: Projections of age-specific incidence and mortality rates and decomposed changes in new cases and deaths — Leukemia; Figure S24: Projections of age-standardized incidence and mortality rates by genders for other cancer sites Figure S25: Projections of new cancer cases by genders for all cancer sites Figure S26: Sensitivity analysis of drift attenuation in ASR in 2050–2054 Figure S27: Projections of new cancer deaths by genders for all cancer sites.
