# Supplementary material for: Long-Term Projections of Cancer Incidence and Mortality in Japan and Decomposition Analysis of Changes in Cancer Burden, 2020–2054: An Empirical Validation Approach
Source: Cancers (Basel). 2022 Dec 9;14(24):6076. doi: 10.3390/cancers14246076 (PMC9775633; doi:10.3390/cancers14246076)
Supplement: Supplementary file 1 [file cancers-14-06076-s001.zip › cancers-2066282-supplementary.pdf]

Supplementary

Title: Long-term projections of cancer incidence and mortality in Japan and decomposition analysis of changes in cancer burden, 2020–2054: An empirical validation approach

Table of Contents

Table S1: Information of empirically selected models with data-, gender- and site-specific parameters ... 2
Table S2: Observed and projected new cancer cases and deaths in 2015–2019 and 2050–2054 by age groups and genders ... 4
Table S3: Cancer incidence by genders and decomposed changes in cases over 2020–2054 for other cancers ... 5
Table S4: Observed and projected top burden of cancer in Japan by genders and age groups ... 6
Table S5: Cancer mortality by genders and decomposed changes in deaths over 2020–2054 for other cancers ... 9
Figure S1: Trend in and projection of total population, proportions of people aged 65+ and 85+, and population pyramids in Japan, 1985–2055 10
Figure S2: Projections of age-specific incidence and mortality rates and decomposed changes in new cases and deaths — Stomach ... 11
Figure S3: Projections of age-specific incidence and mortality rates and decomposed changes in new cases and deaths — Colon/rectum ... 12
Figure S4: Projections of age-specific incidence and mortality rates and decomposed changes in new cases and deaths — Liver ... 13
Figure S5: Projections of age-specific incidence and mortality rates and decomposed changes in new cases and deaths — Pancreas ... 14
Figure S6: Projections of age-specific incidence and mortality rates and decomposed changes in new cases and deaths — Lung, trachea ... 15
Figure S7: Projections of age-specific incidence and mortality rates and decomposed changes in new cases and deaths — Prostate ... 16
Figure S8: Projections of age-specific incidence and mortality rates and decomposed changes in new cases and deaths — Female breast ... 17
Figure S9: Projections of age-specific incidence and mortality rates and decomposed changes in new cases and deaths — Cervix uteri ... 18
Figure S10: Projections of age-specific incidence and mortality rates and decomposed changes in new cases and deaths — Corpus uteri ... 19
Figure S11: Projections of age-specific incidence and mortality rates and decomposed changes in new cases and deaths — Esophagus ... 20
Figure S12: Projections of age-specific incidence and mortality rates and decomposed changes in new cases and deaths — Gallbladder and bile ducts ... 21
Figure S13: Projections of age-specific incidence and mortality rates and decomposed changes in new cases and deaths — Bladder ... 22
Figure S14: Projections of age-specific incidence and mortality rates and decomposed changes in new cases and deaths — Kidney and other urinary organs ... 23
Figure S15: Projections of age-specific incidence and mortality rates and decomposed changes in new cases and deaths — Thyroid ... 24
Figure S16: Projections of age-specific incidence and mortality rates and decomposed changes in new cases and deaths — Malignant lymphoma ... 25
Figure S17: Projections of age-specific incidence and mortality rates and decomposed changes in new cases and deaths — Ovary ... 26
Figure S18: Projections of age-specific incidence and mortality rates and decomposed changes in new cases and deaths — Oral cavity and pharynx ... 27
Figure S19: Projections of age-specific incidence and mortality rates and decomposed changes in new cases and deaths — Larynx ... 28
Figure S20: Projections of age-specific incidence and mortality rates and decomposed changes in new cases and deaths — Skin ... 29
Figure S21: Projections of age-specific incidence and mortality rates and decomposed changes in new cases and deaths — Brain, nervous system ... 30
Figure S22: Projections of age-specific incidence and mortality rates and decomposed changes in new cases and deaths — Multiple myeloma .. 31
Figure S23: Projections of age-specific incidence and mortality rates and decomposed changes in new cases and deaths — Leukemia ... 32
Figure S24: Projections of age-standardized incidence and mortality rates by genders for other cancer sites ... 33
Figure S25: Projections of new cancer cases by genders for all cancer sites ... 34
Figure S26: Sensitivity analysis of drift attenuation in ASR in 2050–2054 ... 35
Figure S27: Projections of new cancer deaths by genders for all cancer sites ... 36

Table S1. Information of empirically selected models with data-, gender- and site-specific parameters.

Data	Gender	Cancer group	Cancer site	ICD-10	Model (link function)	Annual attenuating drift (%)	Use of recent trend	Start included age (years)	Absolute relative bias (%)
Incidence	Male	Major	Stomach	C16	Poisson	6%	YES	15	5.58
			Colon/rectum	C18–C20	Power 3	7%	YES	15	0.14
			Liver	C22	Power 3	6%	YES	20	0.03
			Pancreas	C25	Power 4	10%	YES	25	1.45
			Lung, trachea	C33–C34	Poisson	10%	NO	20	0.16
			Prostate	C61	Power 5	10%	YES	40	1.29
		Sub–major	Esophagus	C15	Power 5	6%	NO	35	0.27
			Gallbladder and bile ducts	C23–C24	Power 7	10%	NO	30	0.02
			Bladder	C67	Power 3	10%	YES	25	0.78
			Kidney and other urinary organs	C64–C66 C68	Power 4	10%	YES	20	0.43
			Thyroid	C73	Power 4	9%	NO	20	2.01
			Malignant lymphoma	C81–C85 C96	Power 3	7%	YES	0	0.17
		Others	Oral cavity and pharynx	C00–C14	Power 7	10%	YES	10	0.55
			Larynx	C32	Poisson	10%	YES	35	2.05
			Skin	C43–C44	Power 3	10%	NO	25	1.11
			Brain, nervous system	C70–C72	Power 4	10%	YES	0	0.04
			Multiple myeloma	C88–C90	Power 4	10%	YES	35	0.14
			Leukemia	C91–C95	Poisson	10%	YES	0	0.82
	Female	Major	Stomach	C16	Poisson	10%	YES	15	2.47
			Colon/rectum	C18–C20	Poisson	10%	YES	15	0.06
			Liver	C22	Power 7	10%	YES	25	0.22
			Pancreas	C25	Power 3	10%	NO	30	0.66
			Lung, trachea	C33–C34	Power 4	8%	NO	25	0.11
			Female breast	C50	Poisson	7%	YES	20	0.31
			Cervix uteri	C53	Poisson	10%	YES	20	0.10
			Corpus uteri	C54	Poisson	7%	NO	25	0.37
		Sub–major	Esophagus	C15	Power 3	9%	NO	40	1.64
			Gallbladder and bile ducts	C23–C24	Poisson	10%	YES	30	0.34
			Bladder	C67	Power 3	10%	NO	30	1.24
			Kidney and other urinary organs	C64–C66 C68	Power 3	6%	YES	20	0.01
			Thyroid	C73	Power 3	10%	NO	10	4.71
			Malignant lymphoma	C81–C85 C96	Power 7	8%	YES	0	3.00
			Ovary	C56	Poisson	8%	NO	5	0.11
		Others	Oral cavity and pharynx	C00–C14	Poisson	6%	NO	15	4.71
			Larynx	C32	Power 3	10%	NO	40	6.75
			Skin	C43–C44	Power 3	10%	NO	15	2.18
			Brain, nervous system	C70–C72	Power 3	8%	NO	0	0.16
			Multiple myeloma	C88–C90	Poisson	7%	NO	35	1.28
			Leukemia	C91–C95	Poisson	10%	YES	0	0.19
Mortality	Male	Major	Stomach	C16	Power 4	10%	YES	20	0.08
			Colon/rectum	C18–C20	Power 6	10%	YES	20	0.02

Data	Gender	Cancer group	Cancer site	ICD-10	Model (link function)	Annual attenuating drift (%)	Use of recent trend	Start included age (years)	Absolute relative bias (%)
			Liver	C22	Power 3	10%	YES	25	7.36
			Pancreas	C25	Poisson	10%	NO	25	1.06
			Lung, trachea	C33–C34	Power 6	10%	YES	25	0.33
			Prostate	C61	Power 5	10%	NO	40	0.21
		Sub–major	Esophagus	C15	Power 3	10%	YES	35	0.04
			Gallbladder and bile ducts	C23–C24	Power 3	6%	YES	30	3.62
			Bladder	C67	Power 3	6%	YES	35	3.75
			Kidney and other urinary organs	C64–C66 C68	Power 4	7%	YES	25	0.06
			Thyroid	C73	Power 3	6%	NO	40	0.26
			Malignant lymphoma	C81–C85 C96	Poisson	10%	YES	10	0.11
		Others	Oral cavity and pharynx	C00–C14	Poisson	10%	YES	20	0.02
			Larynx	C32	Power 3	10%	YES	40	3.32
			Skin	C43–C44	Power 3	10%	YES	30	0.07
			Brain, nervous system	C70–C72	Poisson	10%	YES	0	0.02
			Multiple myeloma	C88–C90	Power 3	10%	YES	35	0.06
			Leukemia	C91–C95	Power 4	9%	YES	0	0.02
	Female	Major	Stomach	C16	Power 7	9%	NO	20	0.00
			Colon/rectum	C18–C20	Power 4	6%	YES	20	0.00
			Liver	C22	Power 7	10%	YES	25	2.96
			Pancreas	C25	Power 3	7%	YES	30	0.17
			Lung, trachea	C33–C34	Power 5	8%	NO	25	0.12
			Female breast	C50	Poisson	6%	NO	25	1.73
			Cervix uteri	C53	Power 3	8%	NO	25	0.10
			Corpus uteri	C54	Poisson	6%	YES	35	0.10
		Sub–major	Esophagus	C15	Power 5	10%	YES	40	0.06
			Gallbladder and bile ducts	C23–C24	Power 5	10%	NO	35	0.15
			Bladder	C67	Power 5	6%	YES	40	2.92
			Kidney and other urinary organs	C64–C66 C68	Poisson	8%	YES	30	1.67
			Thyroid	C73	Power 4	7%	NO	40	0.02
			Malignant lymphoma	C81–C85 C96	Poisson	10%	NO	15	1.12
			Ovary	C56	Poisson	10%	YES	15	1.95
		Others	Oral cavity and pharynx	C00–C14	Power 3	8%	NO	25	0.04
			Larynx	C32	Power 4	8%	NO	40	0.11
			Skin	C43–C44	Power 3	10%	YES	30	2.38
			Brain, nervous system	C70–C72	Power 5	6%	YES	0	2.33
			Multiple myeloma	C88–C90	Poisson	10%	YES	40	0.85
			Leukemia	C91–C95	Power 4	6%	YES	0	0.22

Table S2. Observed and projected new cancer cases and deaths in 2015–2019 and 2050–2054 by age groups and genders.

Gender	Age group	Period 2015–2019			Period 2050–2054		
		Case (%)	Death (%)	Population (%)	Case (%)	Death (%)	Population (%)
All	Oldest-old (85+ years)	131201 (14.01%)	96620 (27.21%)	5442300 (4.30%)	284090 (25.88%)	124876 (40.57%)	9764411 (9.75%)
	Middle-old (75–84 years)	277365 (29.61%)	121094 (34.10%)	11992347 (9.47%)	366481 (33.39%)	108585 (35.27%)	14615368 (14.60%)
	Youngest-old (65–74 years)	290573 (31.03%)	89087 (25.09%)	17579346 (13.88%)	237173 (21.61%)	47220 (15.34%)	13529580 (13.51%)
	Middle-adult (40–64 years)	217183 (23.19%)	46082 (12.98%)	42505899 (33.56%)	191468 (17.44%)	25877 (8.41%)	29181164 (29.14%)
	Young-adult (20–39 years)	17937 (1.92%)	1913 (0.54%)	27596730 (21.79%)	15912 (1.45%)	1054 (0.34%)	18589711 (18.56%)
	Adolescent (15–19 years)	721 (0.08%)	77 (0.02%)	5963683 (4.71%)	740 (0.07%)	61 (0.02%)	3943462 (3.94%)
	Children (0–14 years)	1590 (0.17%)	195 (0.05%)	15589044 (12.31%)	1702 (0.16%)	167 (0.05%)	10510108 (10.50%)
Male	Oldest-old (85+ years)	63836 (11.95%)	46833 (22.23%)	1666348 (2.70%)	139971 (24.51%)	62299 (35.44%)	3407040 (7.04%)
	Middle-old (75–84 years)	174975 (32.75%)	76752 (36.44%)	5145840 (8.35%)	217994 (38.17%)	67945 (38.65%)	6658062 (13.76%)
	Youngest-old (65–74 years)	189144 (35.40%)	59998 (28.48%)	8381733 (13.60%)	135917 (23.80%)	30603 (17.41%)	6586967 (13.62%)
	Middle-adult (40–64 years)	100460 (18.80%)	26132 (12.41%)	21316860 (34.58%)	71788 (12.57%)	14470 (8.23%)	14813800 (30.62%)
	Young-adult (20–39 years)	4731 (0.89%)	786 (0.37%)	14085399 (22.85%)	4035 (0.71%)	360 (0.20%)	9507695 (19.65%)
	Adolescent (15–19 years)	317 (0.06%)	45 (0.02%)	3062278 (4.97%)	374 (0.07%)	35 (0.02%)	2019199 (4.17%)
	Children (0–14 years)	863 (0.16%)	107 (0.05%)	7982089 (12.95%)	984 (0.17%)	84 (0.05%)	5386595 (11.13%)
Female	Oldest-old (85+ years)	67365 (16.75%)	49787 (34.47%)	3775952 (5.81%)	144119 (27.37%)	62576 (47.39%)	6357370 (12.28%)
	Middle-old (75–84 years)	102390 (25.45%)	44342 (30.70%)	6846507 (10.53%)	148487 (28.20%)	40639 (30.78%)	7957305 (15.38%)
	Youngest-old (65–74 years)	101430 (25.22%)	29090 (20.14%)	9197614 (14.14%)	101256 (19.23%)	16617 (12.58%)	6942612 (13.41%)
	Middle-adult (40–64 years)	116723 (29.02%)	19950 (13.81%)	21189038 (32.58%)	119680 (22.73%)	11407 (8.64%)	14367367 (27.76%)
	Young-adult (20–39 years)	13205 (3.28%)	1128 (0.78%)	13511331 (20.78%)	11877 (2.26%)	694 (0.53%)	9082015 (17.55%)
	Adolescent (15–19 years)	404 (0.10%)	32 (0.02%)	2901405 (4.46%)	366 (0.07%)	26 (0.02%)	1924262 (3.72%)
	Children (0–14 years)	727 (0.18%)	88 (0.06%)	7606954 (11.70%)	718 (0.14%)	83 (0.06%)	5123514 (9.90%)

%: Proportion to the total number of all-age combined.

Table S3. Cancer incidence by genders and decomposed changes in cases over 2020–2054 for other cancers.

Gender	Cancer Site	ICD-10	Period 2015–2019[a]			Period 2050–2054[a]			Case changes over period 2020–2054			
			Case	Rate[b]	Rank	Case	Rate[b]	Rank	Total (%)	Population risk (%)[c]	Population age structure (%)[d]	Population size (%)[e]
Male and female combined	Oral cavity and pharynx	C00–C14	21660	9.4	14	31140	13.2	10	+9480 (+43.8%)	+9360 (+43.2%)	+6485 (+29.9%)	-6364 (-29.4%)
	Larynx	C32	5267	2.0	22	3973	1.4	22	-1294 (-24.6%)	-1620 (-30.8%)	+1445 (+27.4%)	-1119 (-21.2%)
	Skin	C43–C44	23727	7.7	11	30508	7.5	11	+6781 (+28.6%)	+832 (+3.5%)	+12446 (+52.5%)	-6497 (-27.4%)
	Brain, nervous system	C70–C72	5913	3.4	21	6980	4.5	21	+1067 (+18.0%)	+1576 (+26.7%)	+1041 (+17.6%)	-1550 (-26.2%)
	Multiple myeloma	C88–C90	7578	2.6	20	10580	3.5	19	+3002 (+39.6%)	+2178 (+28.7%)	+3004 (+39.6%)	-2180 (-28.8%)
	Leukemia	C91–C95	13717	7.1	17	16662	9.1	18	+2945 (+21.5%)	+3243 (+23.6%)	+3357 (+24.5%)	-3655 (-26.6%)
Male	Oral cavity and pharynx	C00–C14	15063	12.6	11	19680	15.6	9	+4617 (+30.7%)	+4616 (+30.6%)	+4255 (+28.2%)	-4254 (-28.2%)
	Larynx	C32	4859	3.4	15	3443	2.2	18	-1416 (-29.1%)	-1743 (-35.9%)	+1338 (+27.5%)	-1011 (-20.8%)
	Skin	C43–C44	12076	7.8	12	14173	7.1	12	+2097 (+17.4%)	-1103 (-9.1%)	+6453 (+53.4%)	-3253 (-26.9%)
	Brain, nervous system	C70–C72	3177	3.7	18	4237	5.6	17	+1060 (+33.4%)	+1356 (+42.7%)	+623 (+19.6%)	-919 (-28.9%)
	Multiple myeloma	C88–C90	4000	2.8	17	5185	3.3	16	+1185 (+29.6%)	+622 (+15.6%)	+1697 (+42.4%)	-1134 (-28.3%)
	Leukemia	C91–C95	8016	8.1	14	9857	10.4	14	+1841 (+23.0%)	+1890 (+23.6%)	+2156 (+26.9%)	-2205 (-27.5%)
Female	Oral cavity and pharynx	C00–C14	6597	5.6	15	11460	10.0	12	+4863 (+73.7%)	+4743 (+71.9%)	+2230 (+33.8%)	-2110 (-32.0%)
	Larynx	C32	408	0.3	21	529	0.4	21	+121 (+29.7%)	+123 (+30.1%)	+107 (+26.2%)	-108 (-26.5%)
	Skin	C43–C44	11651	7.3	11	16335	7.8	9	+4684 (+40.2%)	+1935 (+16.6%)	+5993 (+51.4%)	-3244 (-27.8%)
	Brain, nervous system	C70–C72	2735	3.1	20	2742	3.3	20	+7 (+0.3%)	+220 (+8.0%)	+418 (+15.3%)	-631 (-23.1%)
	Multiple myeloma	C88–C90	3578	2.5	19	5395	3.8	19	+1817 (+50.8%)	+1556 (+43.5%)	+1307 (+36.5%)	-1046 (-29.2%)
	Leukemia	C91–C95	5701	5.9	16	6805	7.6	15	+1104 (+19.4%)	+1353 (+23.7%)	+1201 (+21.1%)	-1450 (-25.4%)

[a]Estimated as annual average of 5-year periods
[b]Age-standardized rate
[c]Changes due to epidemiological factors (i.e., changes in population risks of being diagnosed with or died from cancers, improvements in diagnostic or treatment practices)
[d]Changes due to population age structure
[e]Changes due to population size
%: Proportion to the number of cases in 2015–2019.

Table S4. Observed and projected top burden of cancer in Japan by genders and age groups.

Data	Gender	Age Group	Burden Rank	Period 2015–2019[a]			Period 2050–2054[a]		
				Cancer Site	ICD-10	Case/Death (%)	Cancer Site	ICD-10	Case/Death (%)
Incidence	Male	All age	1	Prostate	C61	89466 (16.7%)	Prostate	C61	114306 (20.0%)
			2	Stomach	C16	88574 (16.6%)	Colon/rectum	C18–C20	109737 (19.2%)
			3	Colon/rectum	C18–C20	86247 (16.1%)	Lung, trachea	C33–C34	97615 (17.1%)
		Oldest-old (85+ years)	1	Lung, trachea	C33–C34	11423 (17.9%)	Prostate	C61	32077 (22.9%)
			2	Stomach	C16	10583 (16.6%)	Lung, trachea	C33–C34	23839 (17.0%)
			3	Prostate	C61	9615 (15.1%)	Colon/rectum	C18–C20	23202 (16.6%)
		Middle-old (75–84 years)	1	Prostate	C61	32515 (18.6%)	Prostate	C61	48743 (22.4%)
			2	Stomach	C16	31013 (17.7%)	Colon/rectum	C18–C20	38836 (17.8%)
			3	Lung, trachea	C33–C34	28219 (16.1%)	Lung, trachea	C33–C34	38697 (17.8%)
		Youngest-old (65–74 years)	1	Prostate	C61	36354 (19.2%)	Colon/rectum	C18–C20	27801 (20.5%)
			2	Stomach	C16	31628 (16.7%)	Prostate	C61	27218 (20.0%)
			3	Colon/rectum	C18–C20	30554 (16.2%)	Lung, trachea	C33–C34	23817 (17.5%)
		Middle-adult (40–64 years)	1	Colon/rectum	C18–C20	20977 (20.9%)	Colon/rectum	C18–C20	18886 (26.3%)
			2	Stomach	C16	14956 (14.9%)	Lung, trachea	C33–C34	11072 (15.4%)
			3	Lung, trachea	C33–C34	12719 (12.7%)	Prostate	C61	6261 (8.7%)
		Young-adult (20–39 years)	1	Colon/rectum	C18–C20	899 (19.0%)	Colon/rectum	C18–C20	970 (24.0%)
			2	Malignant lymphoma	C81–C85 C96	587 (12.4%)	Malignant lymphoma	C81–C85 C96	595 (14.7%)
			3	Leukemia	C91–C95	574 (12.1%)	Leukemia	C91–C95	515 (12.8%)
		Adolescent (15–19 years)	1	Leukemia	C91–C95	107 (33.8%)	Malignant lymphoma	C81–C85 C96	119 (31.8%)
			2	Malignant lymphoma	C81–C85 C96	73 (23.0%)	Leukemia	C91–C95	111 (29.7%)
			3	Brain, nervous system	C70–C72	59 (18.6%)	Brain, nervous system	C70–C72	60 (16.0%)
		Children (0–14 years)	1	Leukemia	C91–C95	416 (48.2%)	Leukemia	C91–C95	378 (38.4%)
			2	Brain, nervous system	C70–C72	178 (20.6%)	Malignant lymphoma	C81–C85 C96	321 (32.6%)
			3	Malignant lymphoma	C81–C85 C96	153 (17.7%)	Brain, nervous system	C70–C72	213 (21.6%)
	Female	All age	1	Female breast	C50	92901 (23.1%)	Female breast	C50	132332 (25.1%)
			2	Colon/rectum	C18–C20	65657 (16.3%)	Colon/rectum	C18–C20	97698 (18.6%)
			3	Lung, trachea	C33–C34	40814 (10.1%)	Lung, trachea	C33–C34	48533 (9.2%)
		Oldest-old (85+ years)	1	Colon/rectum	C18–C20	13828 (20.5%)	Colon/rectum	C18–C20	31970 (22.2%)
			2	Stomach	C16	9101 (13.5%)	Female breast	C50	25094 (17.4%)
			3	Lung, trachea	C33–C34	7877 (11.7%)	Lung, trachea	C33–C34	17908 (12.4%)
		Middle-old (75–84 years)	1	Colon/rectum	C18–C20	19806 (19.3%)	Female breast	C50	31754 (21.4%)
			2	Female breast	C50	14506 (14.2%)	Colon/rectum	C18–C20	30471 (20.5%)
			3	Stomach	C16	13586 (13.3%)	Lung, trachea	C33–C34	17104 (11.5%)
		Youngest-old (65–74 years)	1	Female breast	C50	22702 (22.4%)	Female breast	C50	24986 (24.7%)
			2	Colon/rectum	C18–C20	17706 (17.5%)	Colon/rectum	C18–C20	19892 (19.6%)
			3	Lung, trachea	C33–C34	12765 (12.6%)	Lung, trachea	C33–C34	9031 (8.9%)
		Middle-adult (40–64 years)	1	Female breast	C50	45392 (38.9%)	Female breast	C50	45448 (38.0%)
			2	Colon/rectum	C18–C20	13549 (11.6%)	Corpus uteri	C54	18232 (15.2%)
			3	Corpus uteri	C54	9398 (8.1%)	Colon/rectum	C18–C20	14781 (12.4%)

Data	Gender	Age Group	Burden Rank	Period 2015–2019[a]			Period 2050–2054[a]		
				Cancer Site	ICD-10	Case/Death (%)	Cancer Site	ICD-10	Case/Death (%)
		Young-adult (20–39 years)	1	Female breast	C50	3967 (30.0%)	Female breast	C50	5047 (42.5%)
			2	Cervix uteri	C53	1915 (14.5%)	Thyroid	C73	1200 (10.1%)
			3	Thyroid	C73	1899 (14.4%)	Ovary	C56	1050 (8.8%)
		Adolescent (15–19 years)	1	Ovary	C56	94 (23.3%)	Malignant lymphoma	C81–C85 C96	83 (22.7%)
			2	Thyroid	C73	90 (22.3%)	Ovary	C56	76 (20.8%)
			3	Leukemia	C91–C95	73 (18.1%)	Leukemia	C91–C95	68 (18.6%)
		Children (0–14 years)	1	Leukemia	C91–C95	328 (45.1%)	Leukemia	C91–C95	303 (42.2%)
			2	Brain, nervous system	C70–C72	148 (20.4%)	Malignant lymphoma	C81–C85 C96	171 (23.8%)
			3	Malignant lymphoma	C81–C85 C96	84 (11.6%)	Brain, nervous system	C70–C72	121 (16.9%)
Mortality	Male	All age	1	Lung, trachea	C33–C34	52872 (25.1%)	Lung, trachea	C33–C34	42325 (24.1%)
			2	Stomach	C16	29457 (14.0%)	Colon/rectum	C18–C20	26083 (14.8%)
			3	Colon/rectum	C18–C20	27135 (12.9%)	Stomach	C16	19634 (11.2%)
		Oldest-old (85+ years)	1	Lung, trachea	C33–C34	11556 (24.7%)	Lung, trachea	C33–C34	14464 (23.2%)
			2	Stomach	C16	6653 (14.2%)	Colon/rectum	C18–C20	8937 (14.3%)
			3	Colon/rectum	C18–C20	5510 (11.8%)	Prostate	C61	8249 (13.2%)
		Middle-old (75–84 years)	1	Lung, trachea	C33–C34	19734 (25.7%)	Lung, trachea	C33–C34	18063 (26.6%)
			2	Stomach	C16	10887 (14.2%)	Colon/rectum	C18–C20	9428 (13.9%)
			3	Colon/rectum	C18–C20	9046 (11.8%)	Stomach	C16	7197 (10.6%)
		Youngest-old (65–74 years)	1	Lung, trachea	C33–C34	15876 (26.5%)	Lung, trachea	C33–C34	7348 (24.0%)
			2	Stomach	C16	8347 (13.9%)	Colon/rectum	C18–C20	4866 (15.9%)
			3	Colon/rectum	C18–C20	8067 (13.4%)	Stomach	C16	3847 (12.6%)
		Middle-adult (40–64 years)	1	Lung, trachea	C33–C34	5634 (21.6%)	Colon/rectum	C18–C20	2768 (19.1%)
			2	Colon/rectum	C18–C20	4366 (16.7%)	Lung, trachea	C33–C34	2420 (16.7%)
			3	Stomach	C16	3458 (13.2%)	Stomach	C16	2181 (15.1%)
		Young-adult (20–39 years)	1	Colon/rectum	C18–C20	145 (18.4%)	Colon/rectum	C18–C20	83 (23.1%)
			2	Leukemia	C91–C95	124 (15.8%)	Brain, nervous system	C70–C72	77 (21.4%)
			3	Stomach	C16	112 (14.2%)	Leukemia	C91–C95	34 (9.4%)
		Adolescent (15–19 years)	1	Leukemia	C91–C95	20 (44.4%)	Brain, nervous system	C70–C72	15 (42.9%)
			2	Brain, nervous system	C70–C72	14 (31.1%)	Leukemia	C91–C95	10 (28.6%)
			3	Malignant lymphoma	C81–C85 C96	4 (8.9%)	Malignant lymphoma	C81–C85 C96	5 (14.3%)
		Children (0–14 years)	1	Brain, nervous system	C70–C72	48 (44.9%)	Brain, nervous system	C70–C72	46 (54.8%)
			2	Leukemia	C91–C95	42 (39.3%)	Leukemia	C91–C95	26 (31.0%)
			3	Malignant lymphoma	C81–C85 C96	6 (5.6%)	Malignant lymphoma	C81–C85 C96	5 (6.0%)
	Female	All age	1	Colon/rectum	C18–C20	23373 (16.2%)	Colon/rectum	C18–C20	24654 (18.7%)
			2	Lung, trachea	C33–C34	21535 (14.9%)	Lung, trachea	C33–C34	19655 (14.9%)
			3	Pancreas	C25	16920 (11.7%)	Pancreas	C25	19545 (14.8%)
		Oldest-old (85+ years)	1	Colon/rectum	C18–C20	9382 (18.8%)	Colon/rectum	C18–C20	13137 (21.0%)
			2	Lung, trachea	C33–C34	7609 (15.3%)	Lung, trachea	C33–C34	9852 (15.7%)
			3	Stomach	C16	6341 (12.7%)	Pancreas	C25	9001 (14.4%)
		Middle-old (75–84 years)	1	Lung, trachea	C33–C34	7255 (16.4%)	Colon/rectum	C18–C20	7068 (17.4%)
			2	Colon/rectum	C18–C20	6751 (15.2%)	Pancreas	C25	6783 (16.7%)

Data	Gender	Age Group	Burden Rank	Period 2015–2019[a]			Period 2050–2054[a]		
				Cancer Site	ICD-10	Case/Death (%)	Cancer Site	ICD-10	Case/Death (%)
			3	Pancreas	C25	5990 (13.5%)	Lung, trachea	C33–C34	6612 (16.3%)
		Youngest-old (65–74 years)	1	Lung, trachea	C33–C34	4700 (16.2%)	Colon/rectum	C18–C20	2793 (16.8%)
			2	Colon/rectum	C18–C20	4344 (14.9%)	Pancreas	C25	2591 (15.6%)
			3	Pancreas	C25	3880 (13.3%)	Lung, trachea	C33–C34	2235 (13.5%)
		Middle-adult (40–64 years)	1	Female breast	C50	5011 (25.1%)	Female breast	C50	2813 (24.7%)
			2	Colon/rectum	C18–C20	2781 (13.9%)	Colon/rectum	C18–C20	1598 (14.0%)
			3	Lung, trachea	C33–C34	1924 (9.6%)	Pancreas	C25	1132 (9.9%)
		Young-adult (20–39 years)	1	Female breast	C50	268 (23.8%)	Female breast	C50	225 (32.4%)
			2	Cervix uteri	C53	163 (14.5%)	Cervix uteri	C53	97 (14.0%)
			3	Stomach	C16	123 (10.9%)	Brain, nervous system	C70–C72	72 (10.4%)
		Adolescent (15–19 years)	1	Leukemia	C91–C95	14 (43.8%)	Brain, nervous system	C70–C72	13 (50.0%)
			2	Brain, nervous system	C70–C72	7 (21.9%)	Leukemia	C91–C95	7 (26.9%)
			3	Ovary	C56	2 (6.2%)	Stomach	C16	1 (3.8%)
		Children (0–14 years)	1	Brain, nervous system	C70–C72	40 (45.5%)	Brain, nervous system	C70–C72	52 (62.7%)
			2	Leukemia	C91–C95	36 (40.9%)	Leukemia	C91–C95	22 (26.5%)
			3	Liver	C22	5 (5.7%)	Liver	C22	3 (3.6%)

[a]Estimated as annual average of 5-year periods

%: Proportion to the number of all-site combined cases/deaths.

Table S5. Cancer mortality by genders and decomposed changes in deaths over 2020–2054 for other cancers.

Gender	Cancer Site	ICD-10	Period 2015–2019[a]			Period 2050–2054[a]			Death changes over period 2020–2054			
			Death	Rate[b]	Rank	Death	Rate[b]	Rank	Total (%)	Population risk (%)[c]	Population age structure (%)[d]	Population size (%)[e]
Male and female combined	Oral cavity and pharynx	C00–C14	7569	2.6	14	6539	1.8	14	-1030 (-13.6%)	-2305 (-30.5%)	+3021 (+39.9%)	-1746 (-23.1%)
	Larynx	C32	899	0.3	22	745	0.2	22	-154 (-17.1%)	-380 (-42.3%)	+436 (+48.5%)	-210 (-23.4%)
	Skin	C43–C44	1593	0.5	21	1795	0.4	20	+202 (+12.7%)	-311 (-19.5%)	+931 (+58.4%)	-418 (-26.2%)
	Brain, nervous system	C70–C72	2653	1.3	18	3620	2.0	17	+967 (+36.4%)	+1091 (+41.1%)	+648 (+24.4%)	-772 (-29.1%)
	Multiple myeloma	C88–C90	4276	1.2	16	3124	0.8	19	-1152 (-26.9%)	-2065 (-48.3%)	+1837 (+43.0%)	-924 (-21.6%)
	Leukemia	C91–C95	8714	3.1	12	6902	1.9	13	-1812 (-20.8%)	-3176 (-36.4%)	+3282 (+37.7%)	-1918 (-22.0%)
Male	Oral cavity and pharynx	C00–C14	5376	3.7	12	4131	2.3	13	-1245 (-23.2%)	-1971 (-36.7%)	+1923 (+35.8%)	-1198 (-22.3%)
	Larynx	C32	827	0.5	16	656	0.3	17	-171 (-20.7%)	-380 (-45.9%)	+399 (+48.2%)	-191 (-23.1%)
	Skin	C43–C44	791	0.5	17	903	0.4	16	+112 (+14.2%)	-143 (-18.1%)	+472 (+59.7%)	-217 (-27.4%)
	Brain, nervous system	C70–C72	1502	1.5	15	1938	2.0	14	+436 (+29.0%)	+487 (+32.4%)	+385 (+25.6%)	-435 (-29.0%)
	Multiple myeloma	C88–C90	2167	1.2	14	1778	0.8	15	-389 (-18.0%)	-933 (-43.1%)	+1053 (+48.6%)	-509 (-23.5%)
	Leukemia	C91–C95	5270	3.7	13	4342	2.3	12	-928 (-17.6%)	-1839 (-34.9%)	+2124 (+40.3%)	-1212 (-23.0%)
Female	Oral cavity and pharynx	C00–C14	2193	1.3	15	2408	1.1	16	+215 (+9.8%)	-334 (-15.2%)	+1098 (+50.1%)	-549 (-25.0%)
	Larynx	C32	72	0.0	21	89	0.0	21	+17 (+23.6%)	-0 (-0.0%)	+36 (+50.0%)	-19 (-26.4%)
	Skin	C43–C44	802	0.4	20	891	0.3	20	+89 (+11.1%)	-168 (-20.9%)	+459 (+57.2%)	-201 (-25.1%)
	Brain, nervous system	C70–C72	1151	1.1	19	1681	1.9	17	+530 (+46.0%)	+604 (+52.5%)	+263 (+22.8%)	-336 (-29.2%)
	Multiple myeloma	C88–C90	2110	1.2	16	1346	0.7	18	-764 (-36.2%)	-1132 (-53.6%)	+784 (+37.2%)	-416 (-19.7%)
	Leukemia	C91–C95	3444	2.5	10	2560	1.4	15	-884 (-25.7%)	-1337 (-38.8%)	+1158 (+33.6%)	-705 (-20.5%)

[a]Estimated as annual average of 5-year periods
[b]Age-standardized rate
[c]Changes due to epidemiological factors (i.e., changes in population risks of being diagnosed with or died from cancers, improvements in diagnostic or treatment practices)
[d]Changes due to population age structure
[e]Changes due to population size
%: Proportion to the number of deaths in 2015–2019.

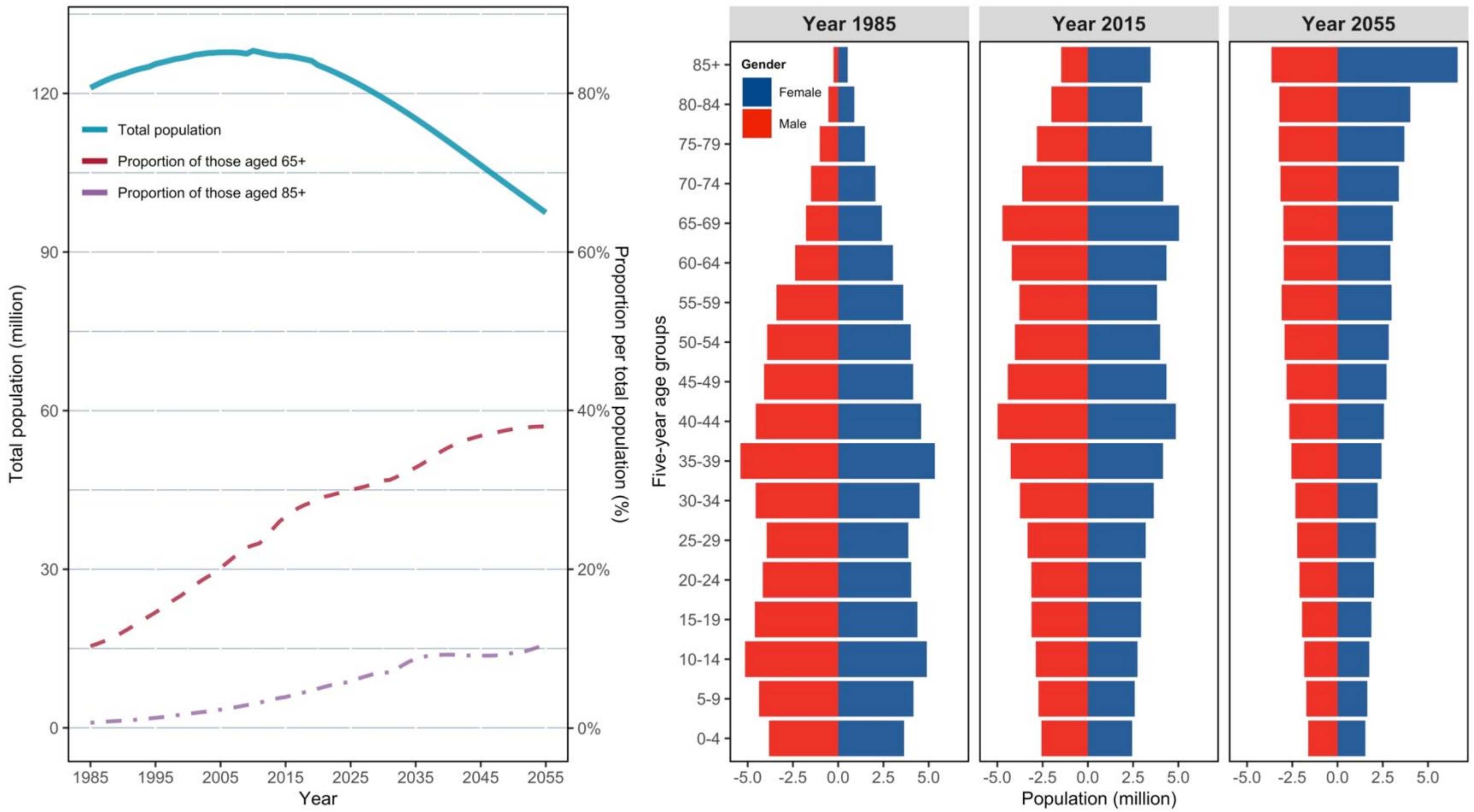

Figure S1. Trend in and projection of total population, proportions of people aged 65+ and 85+, and population pyramids in Japan, 1985–2055.

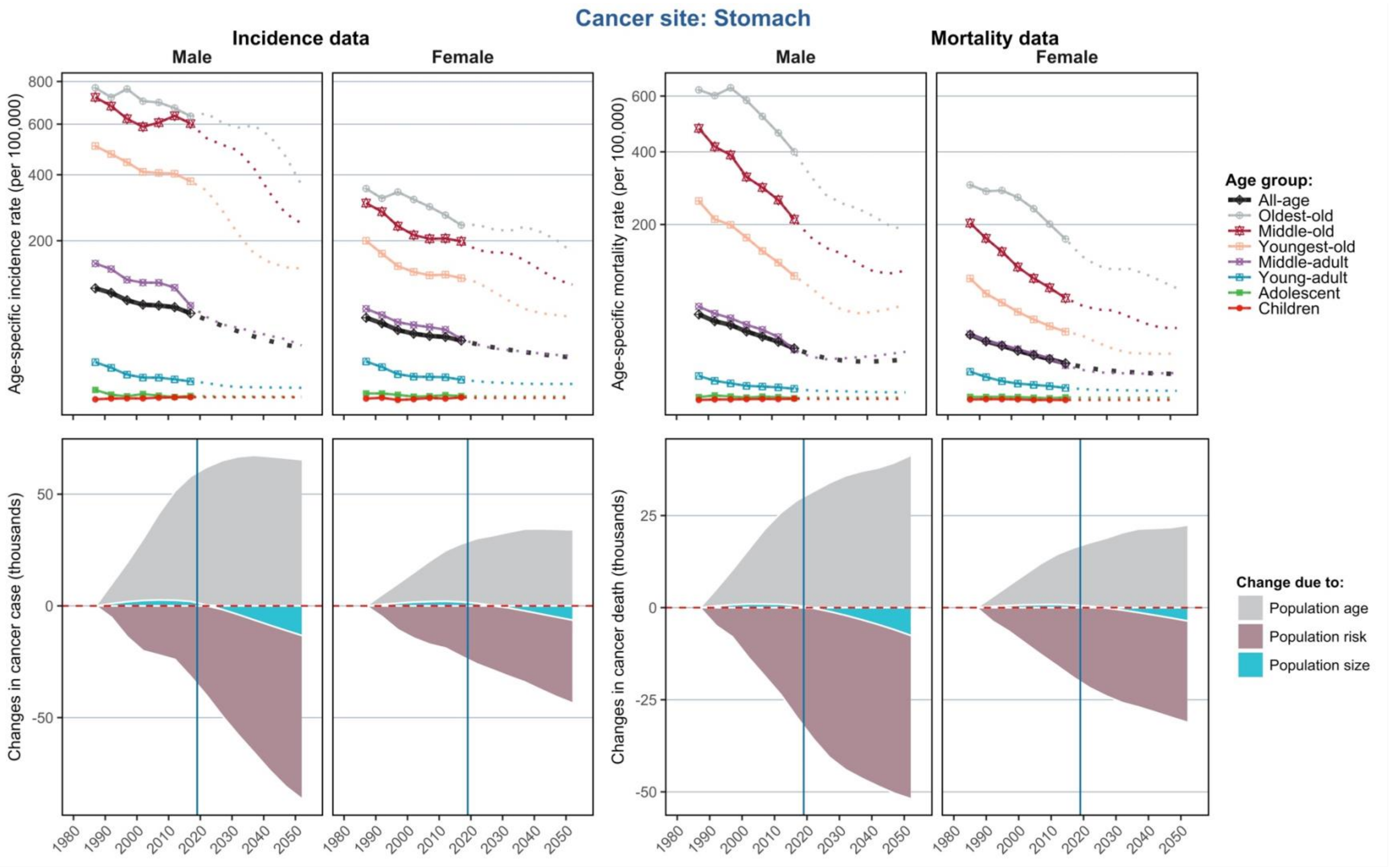

Figure S2. Projections of age-specific incidence and mortality rates and decomposed changes in new cases and deaths — Stomach.

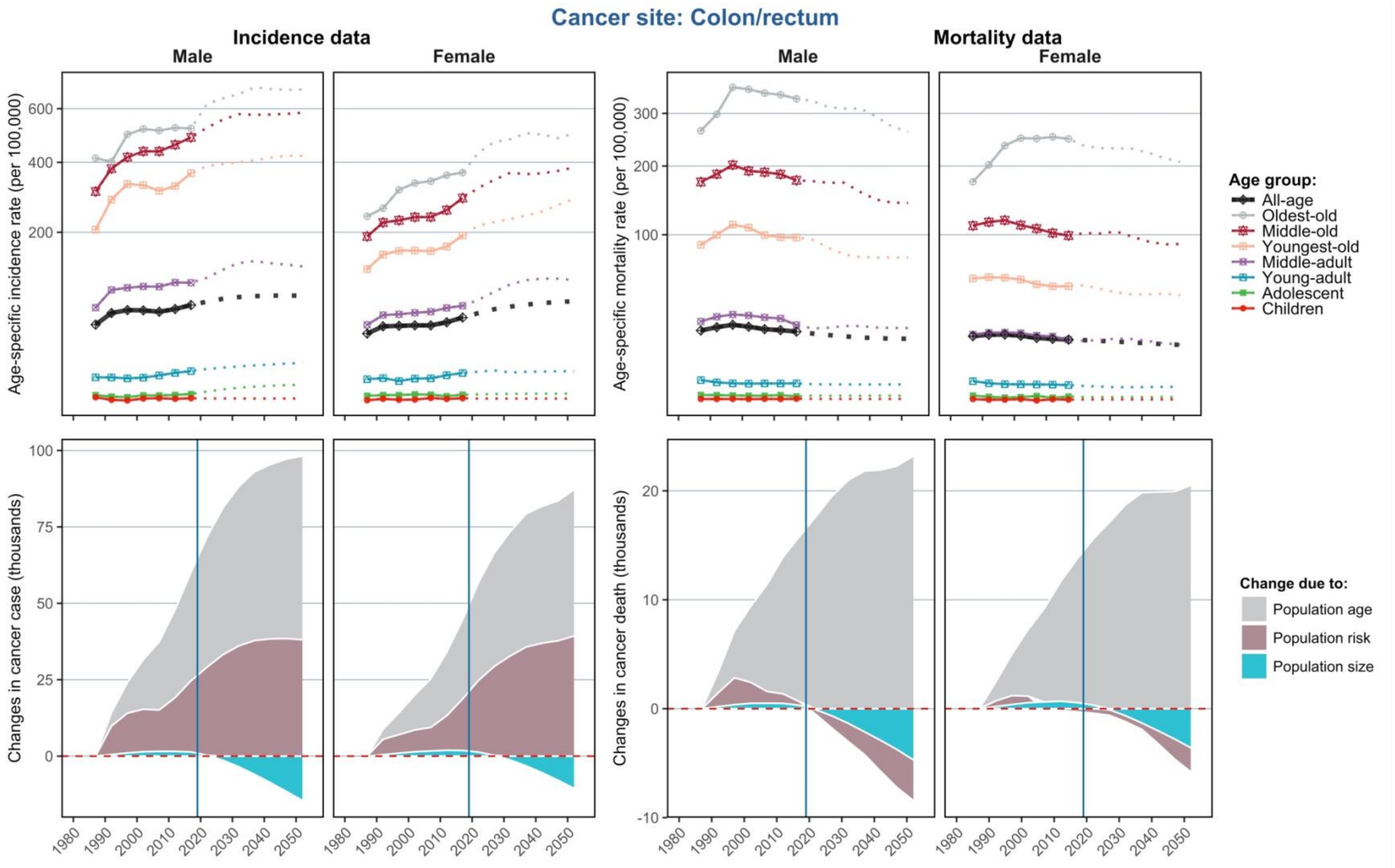

Figure S3. Projections of age-specific incidence and mortality rates and decomposed changes in new cases and deaths — Colon/rectum.

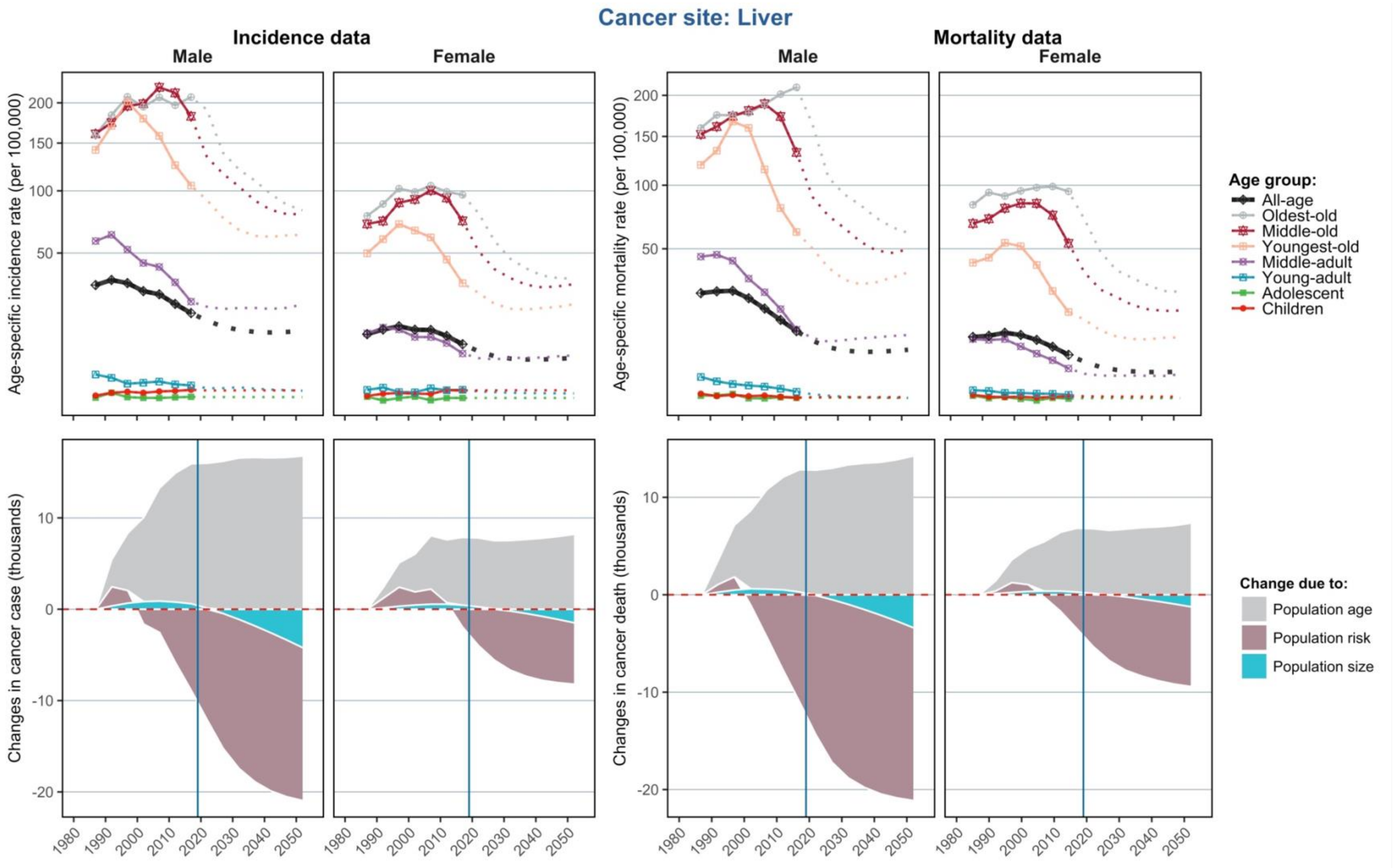

Figure S4. Projections of age-specific incidence and mortality rates and decomposed changes in new cases and deaths — Liver.

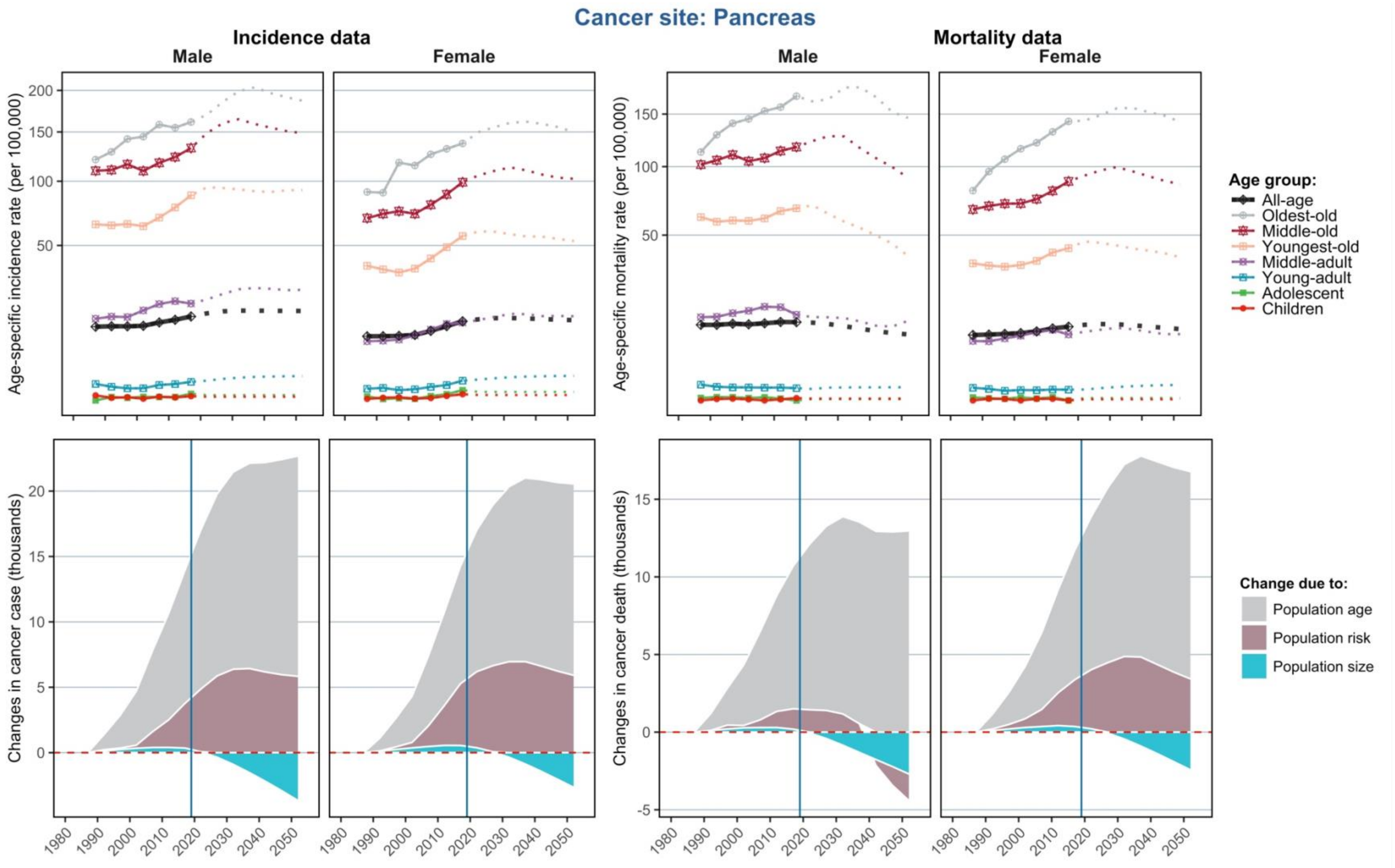

Figure S5. Projections of age-specific incidence and mortality rates and decomposed changes in new cases and deaths — Pancreas.

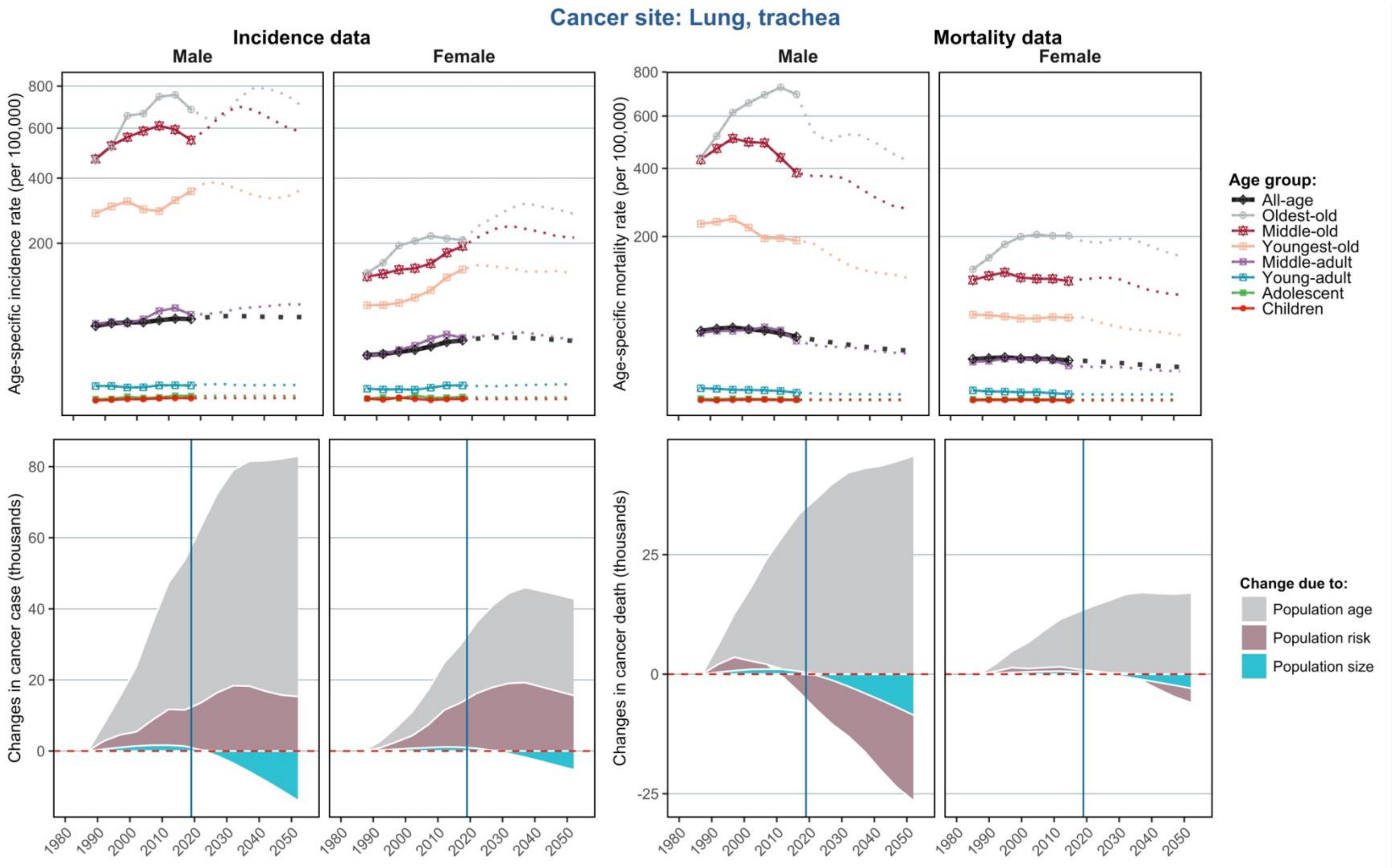

Figure S6. Projections of age-specific incidence and mortality rates and decomposed changes in new cases and deaths — Lung, trachea.

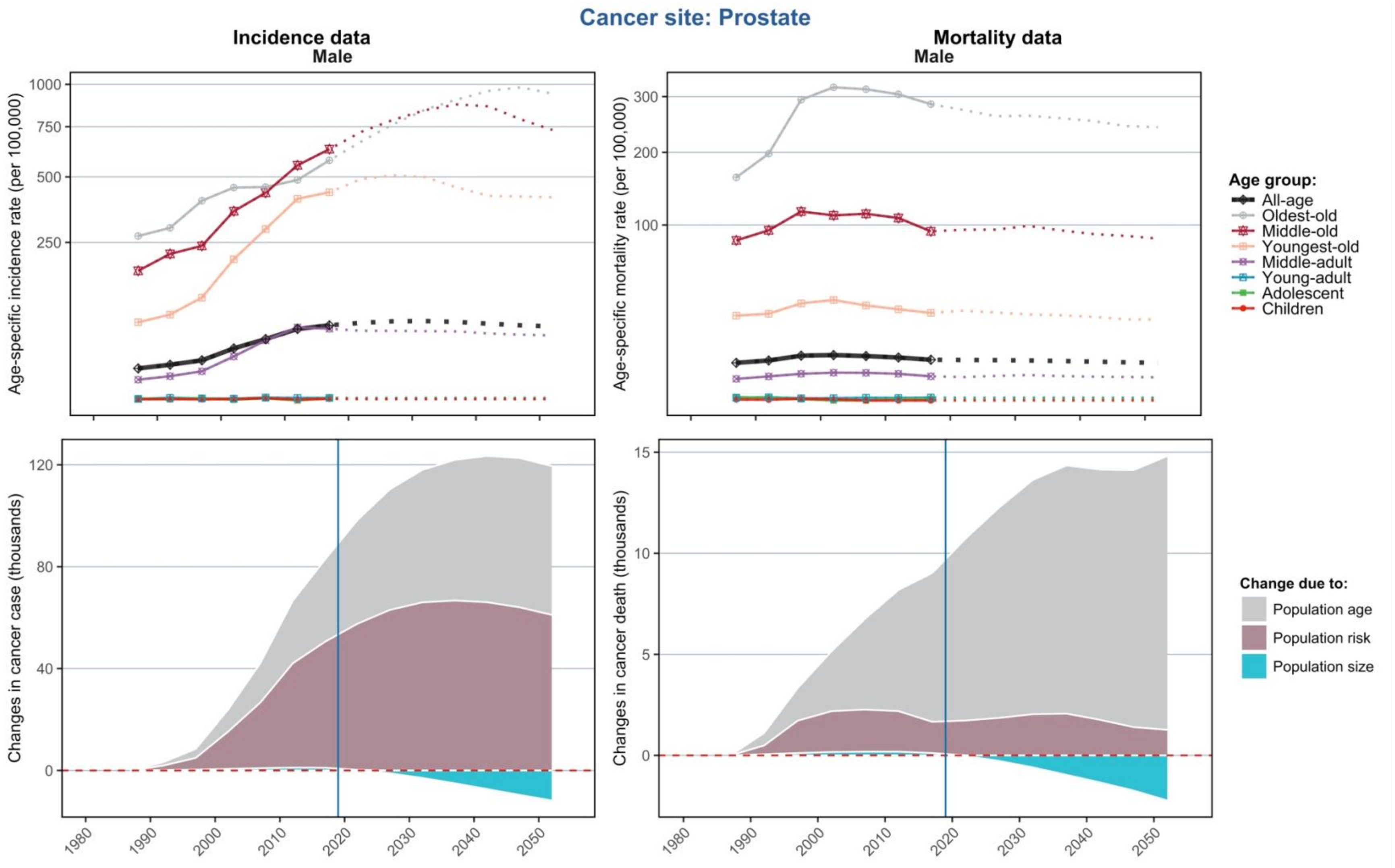

Figure S7. Projections of age-specific incidence and mortality rates and decomposed changes in new cases and deaths — Prostate.

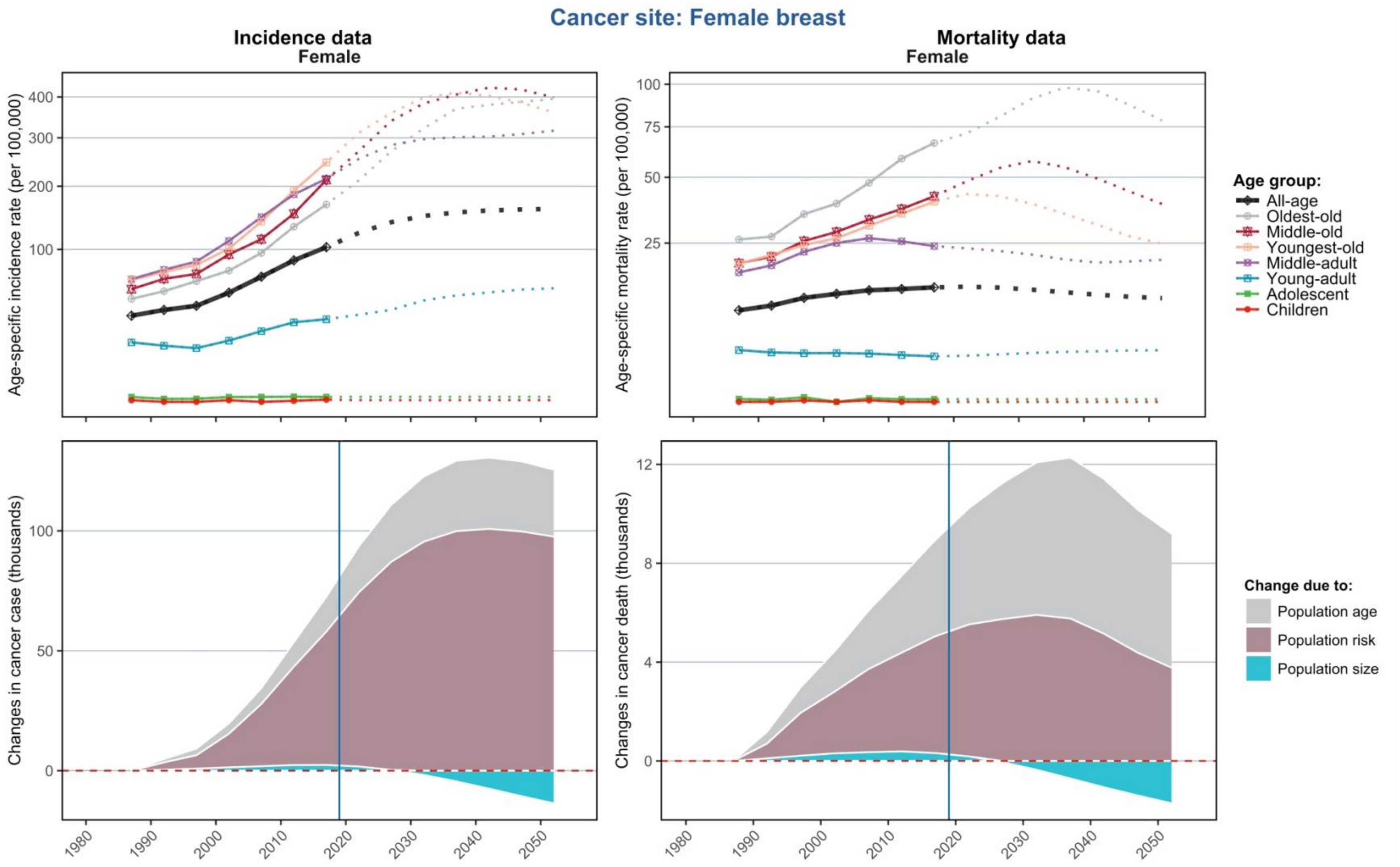

Figure S8. Projections of age-specific incidence and mortality rates and decomposed changes in new cases and deaths — Female breast.

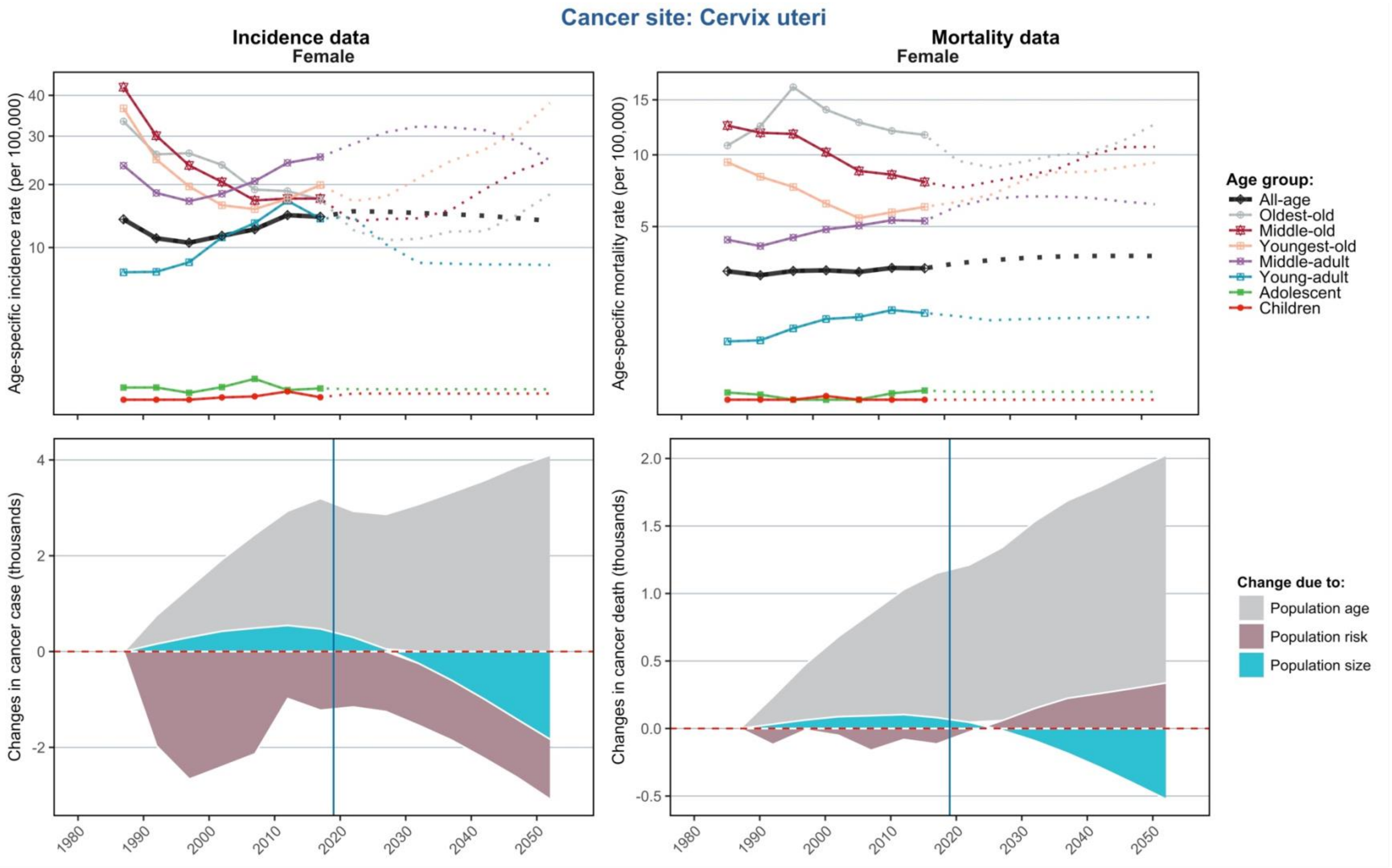

Figure S9. Projections of age-specific incidence and mortality rates and decomposed changes in new cases and deaths — Cervix uteri.

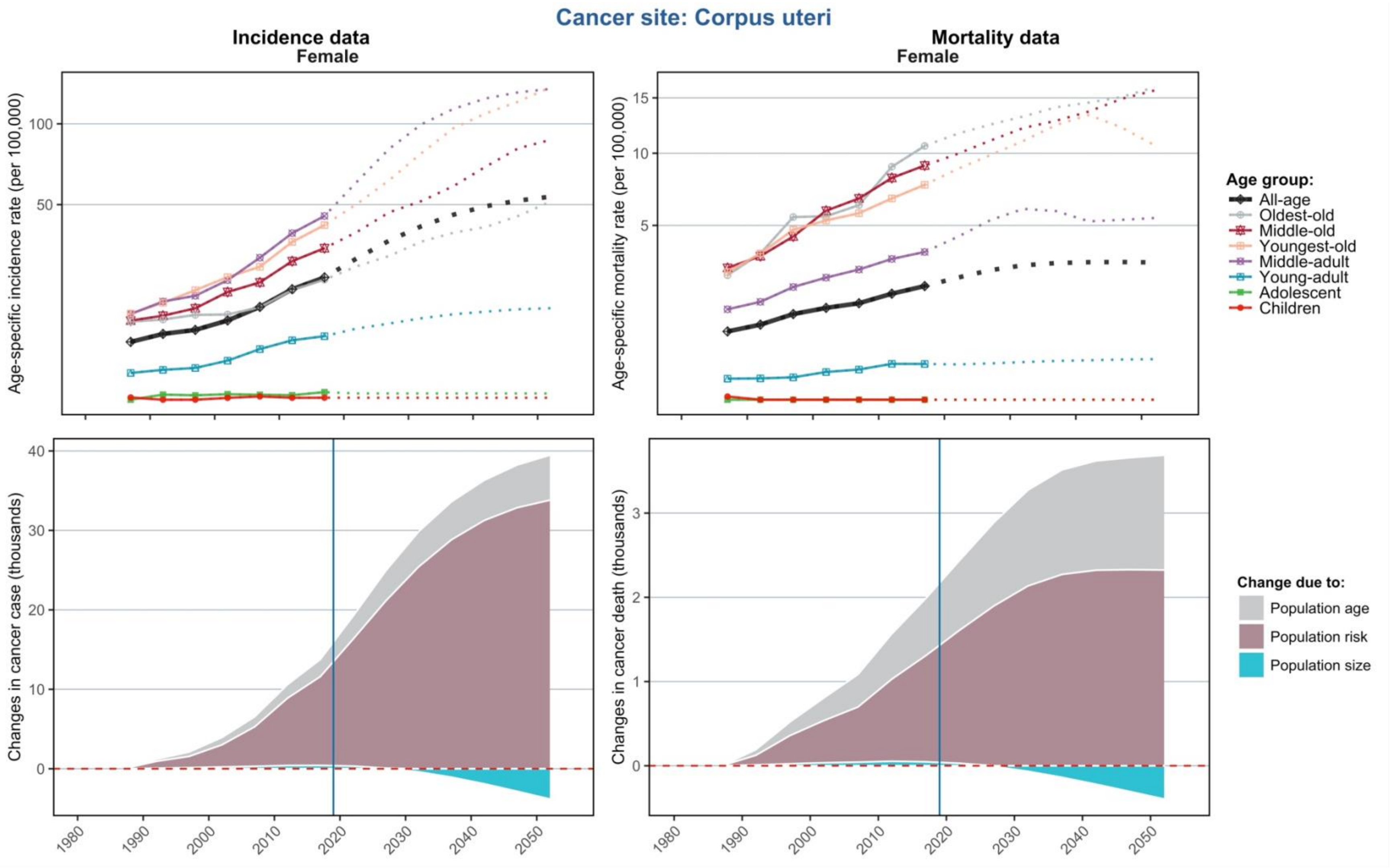

Figure S10. Projections of age-specific incidence and mortality rates and decomposed changes in new cases and deaths — Corpus uteri.

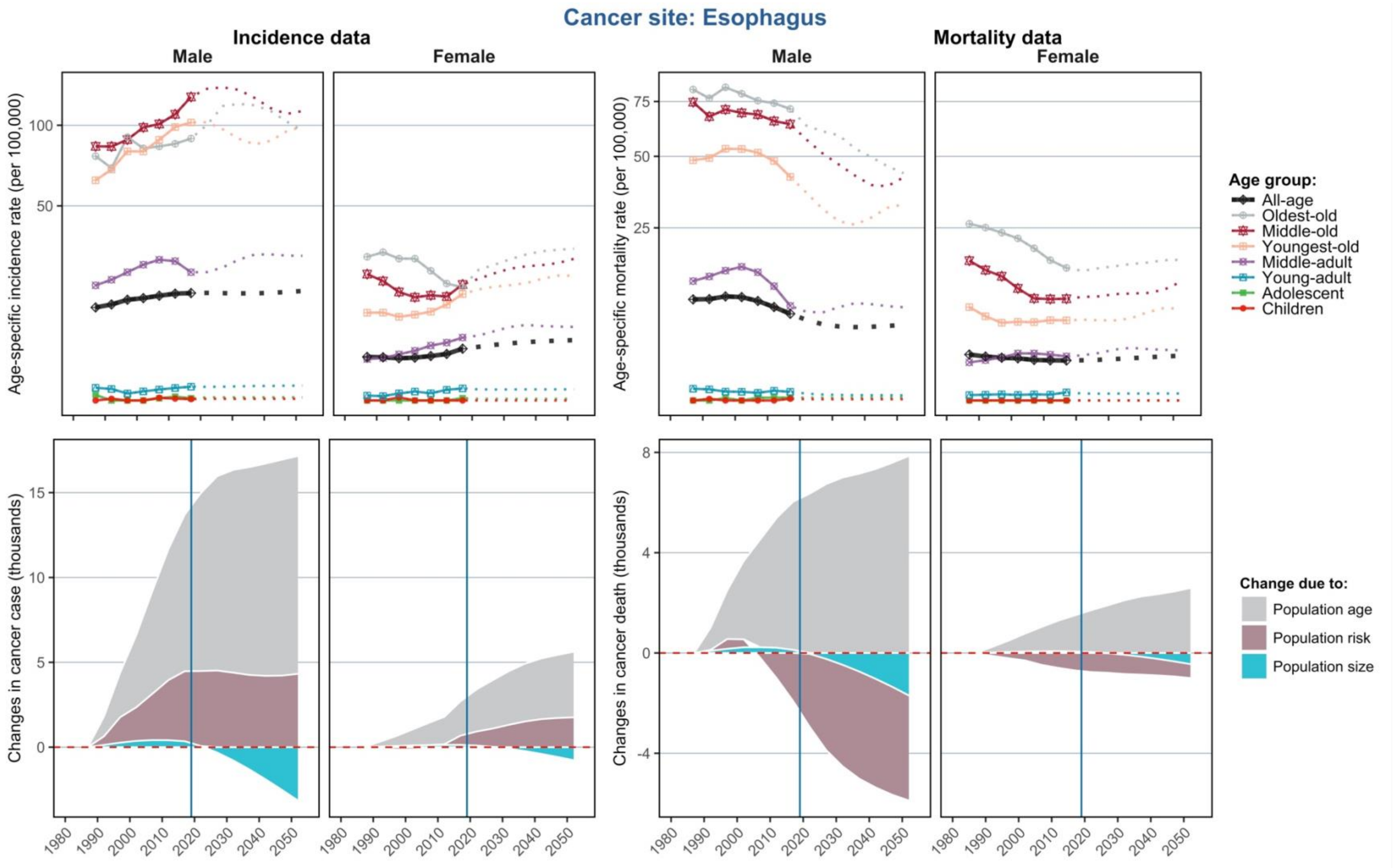

Figure S11. Projections of age-specific incidence and mortality rates and decomposed changes in new cases and deaths — Esophagus.

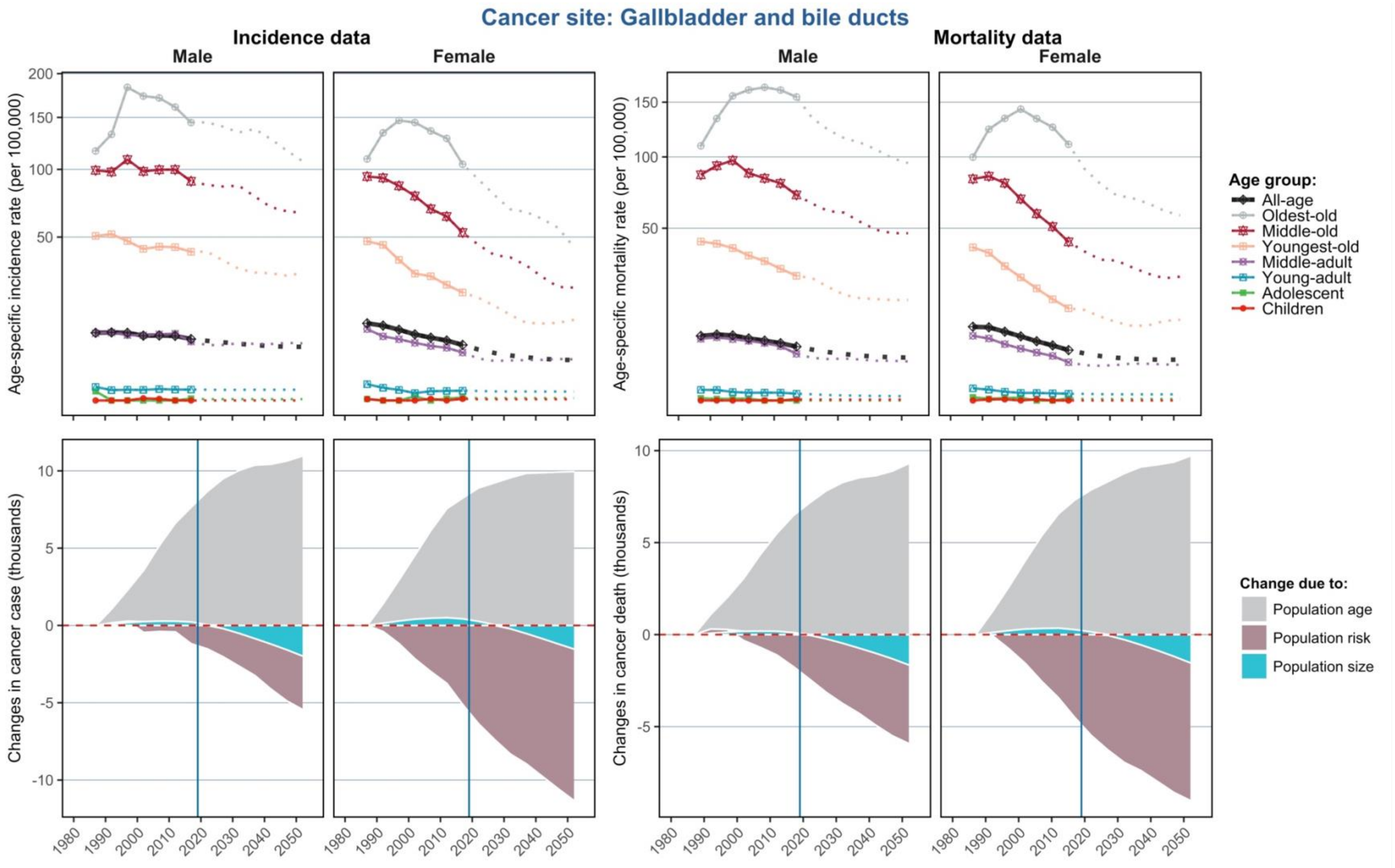

Figure S12. Projections of age-specific incidence and mortality rates and decomposed changes in new cases and deaths — Gallbladder and bile ducts.

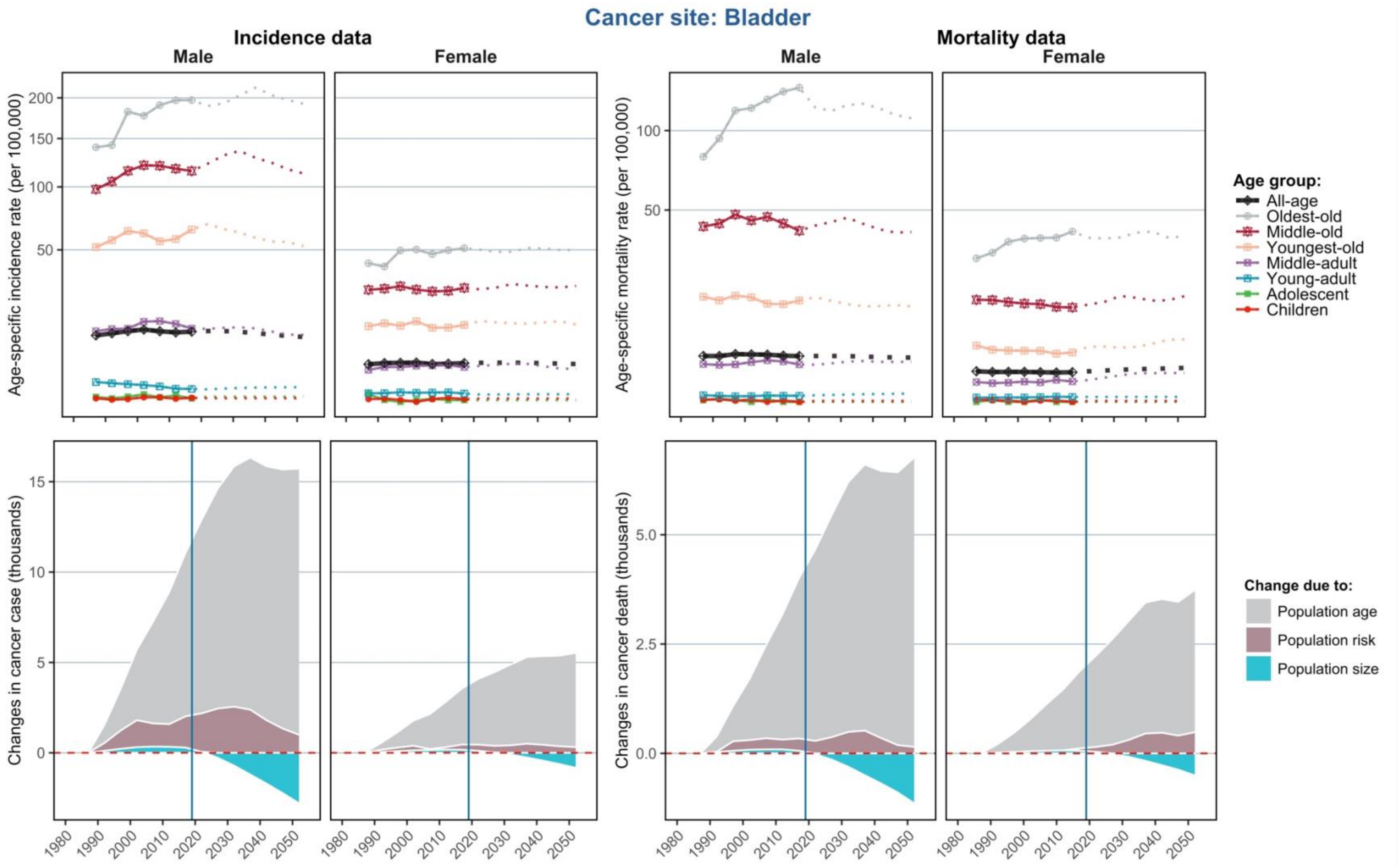

Figure S13. Projections of age-specific incidence and mortality rates and decomposed changes in new cases and deaths — Bladder.

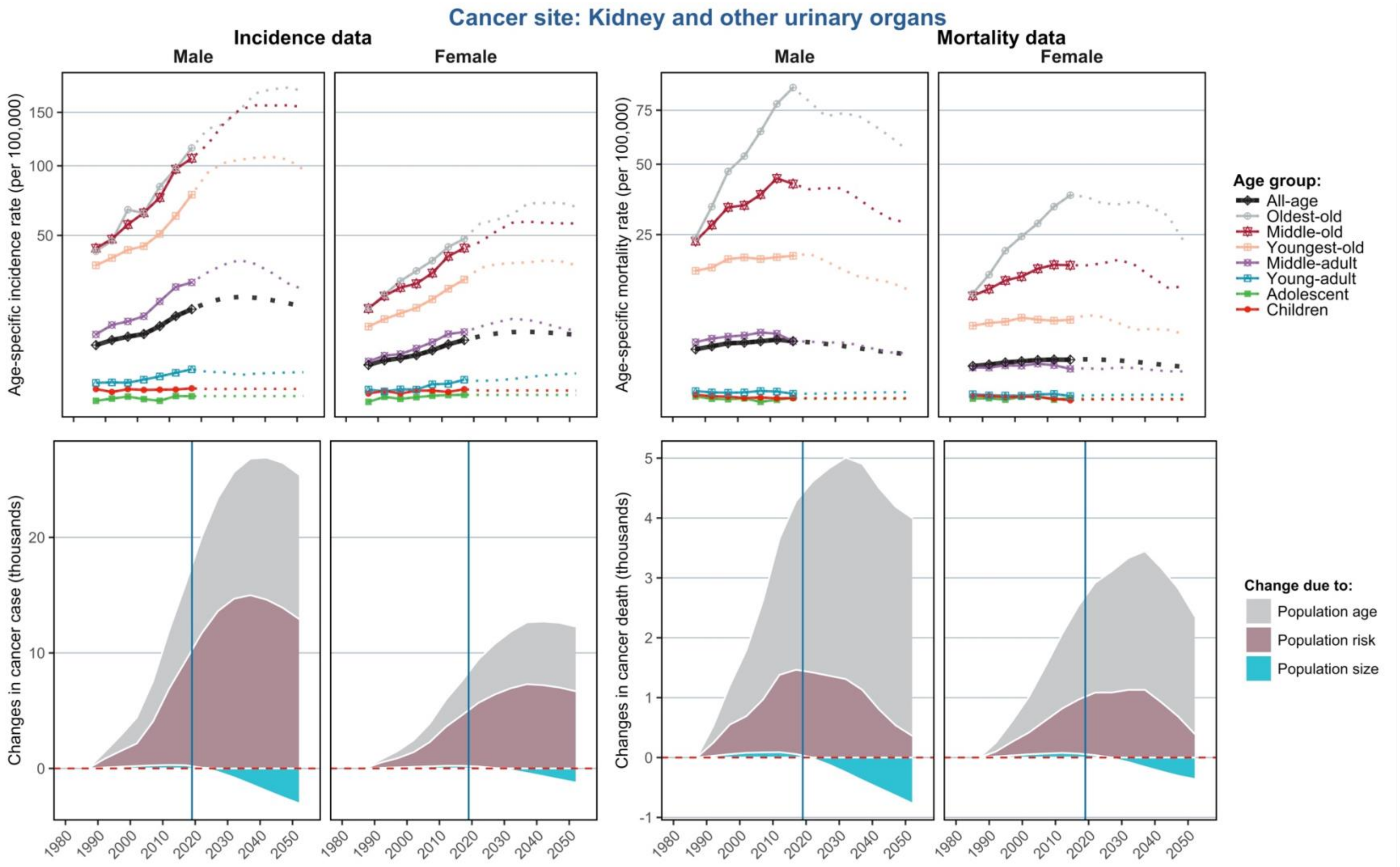

Figure S14. Projections of age-specific incidence and mortality rates and decomposed changes in new cases and deaths — Kidney and other urinary organs.

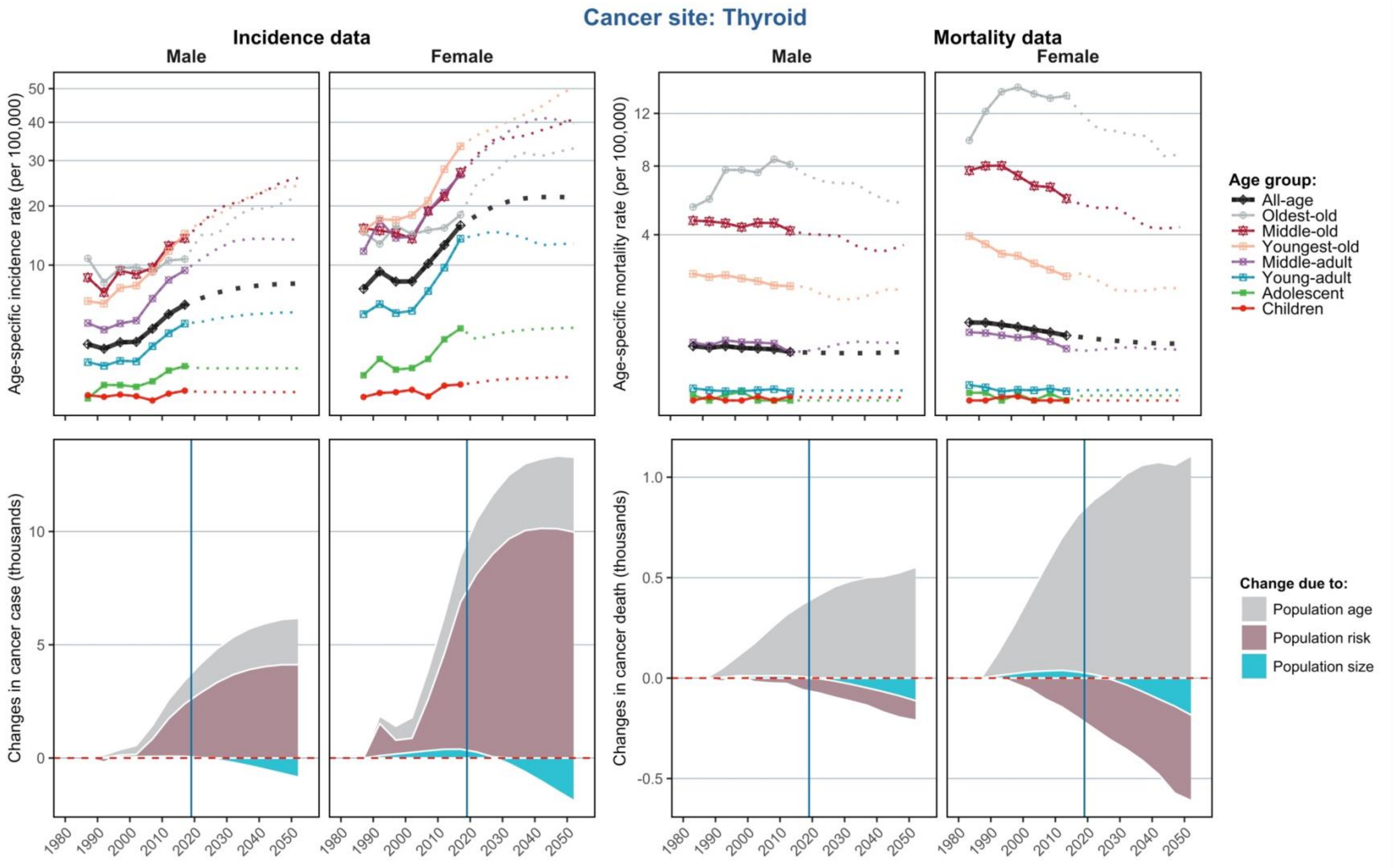

Figure S15. Projections of age-specific incidence and mortality rates and decomposed changes in new cases and deaths — Thyroid.

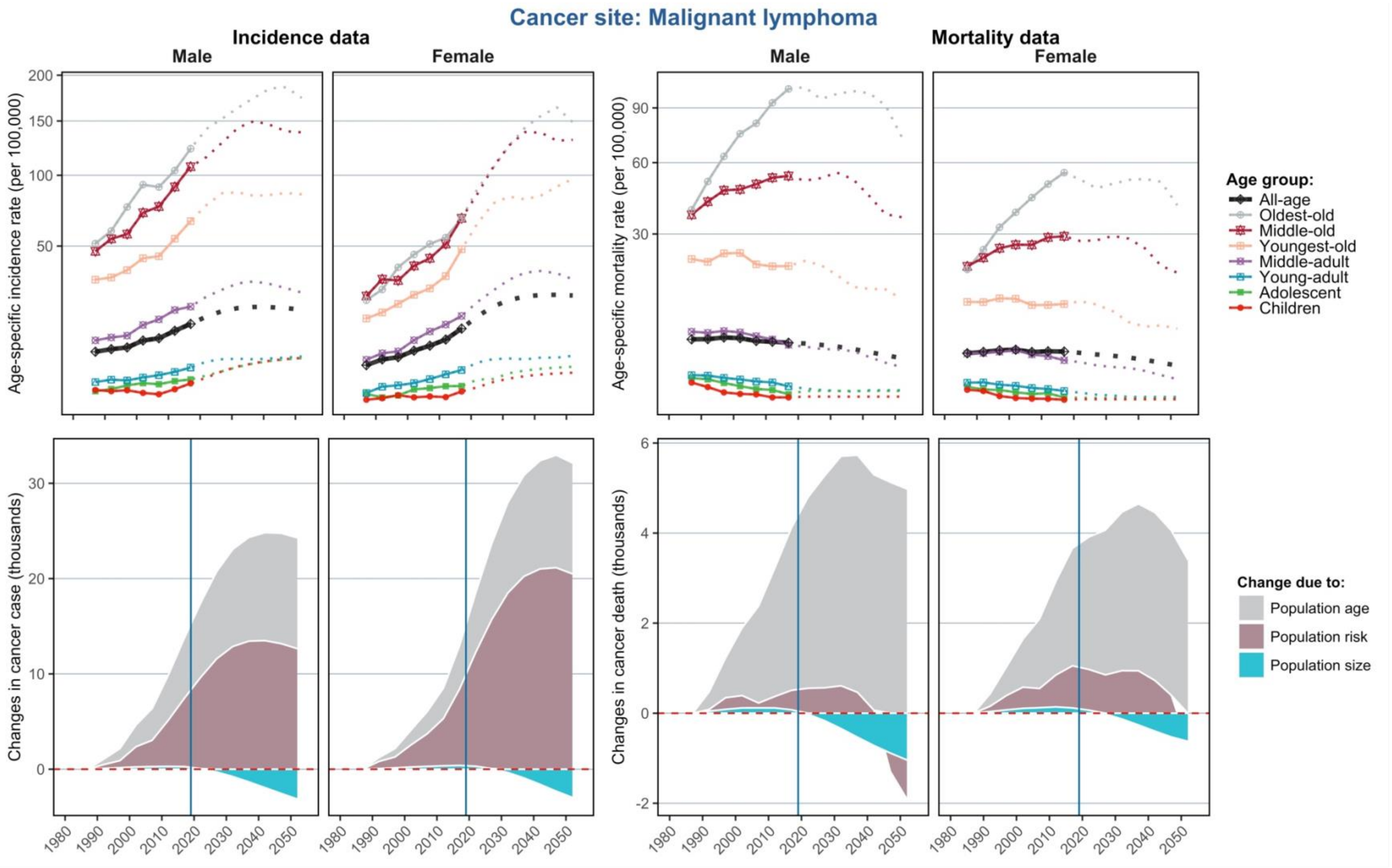

Figure S16. Projections of age-specific incidence and mortality rates and decomposed changes in new cases and deaths — Malignant lymphoma.

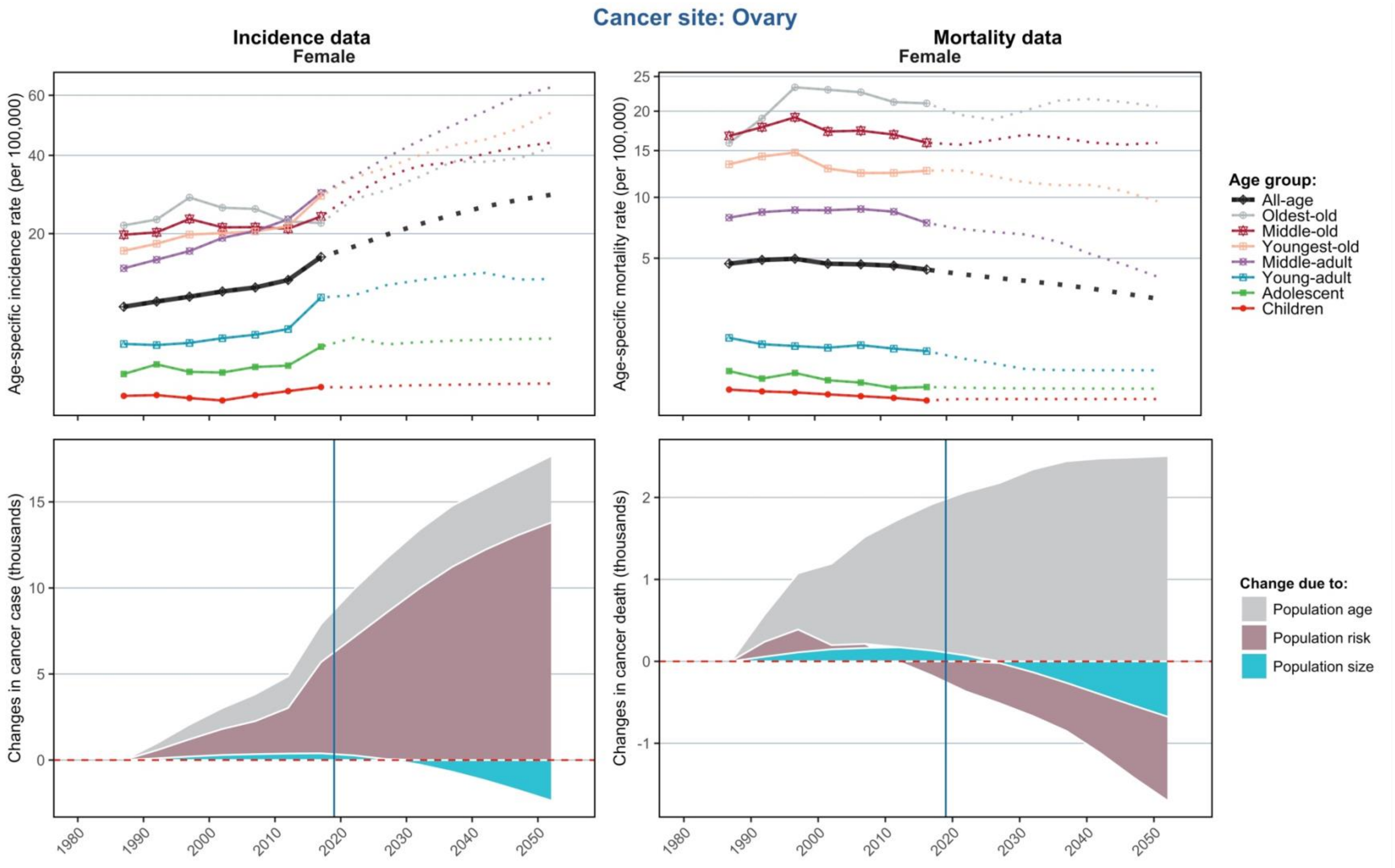

Figure S17. Projections of age-specific incidence and mortality rates and decomposed changes in new cases and deaths — Ovary.

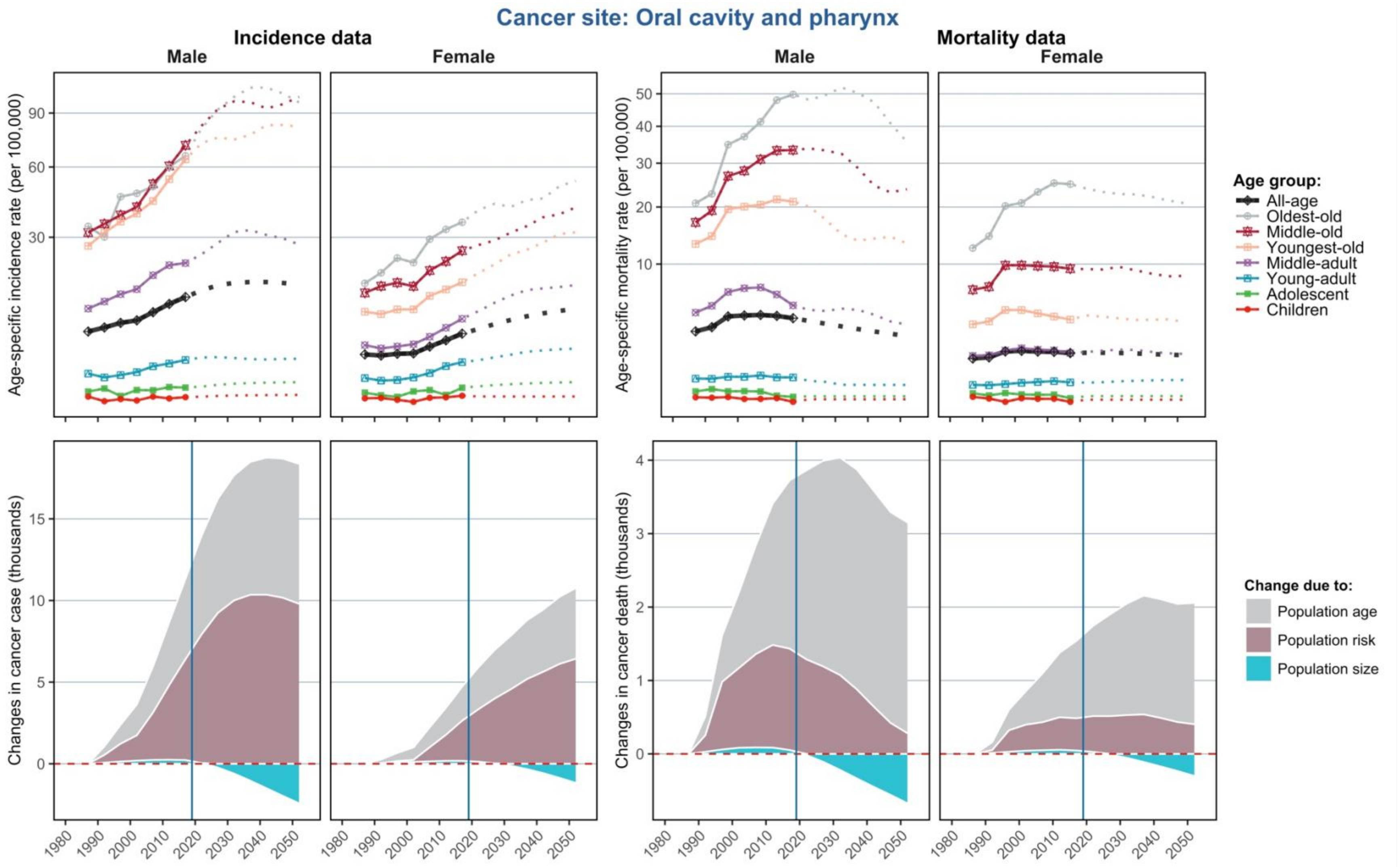

Figure S18. Projections of age-specific incidence and mortality rates and decomposed changes in new cases and deaths — Oral cavity and pharynx.

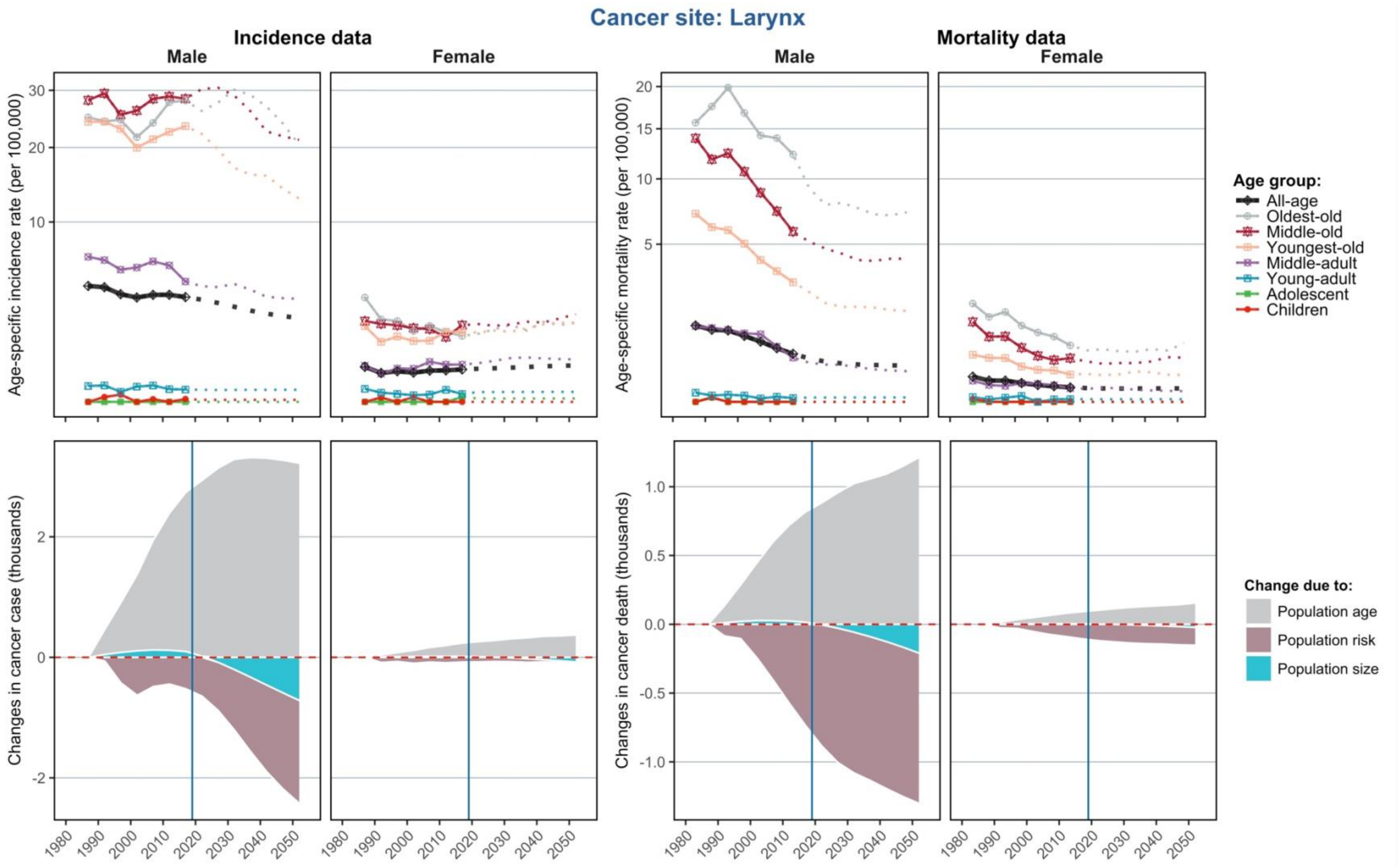

Figure S19. Projections of age-specific incidence and mortality rates and decomposed changes in new cases and deaths — Larynx.

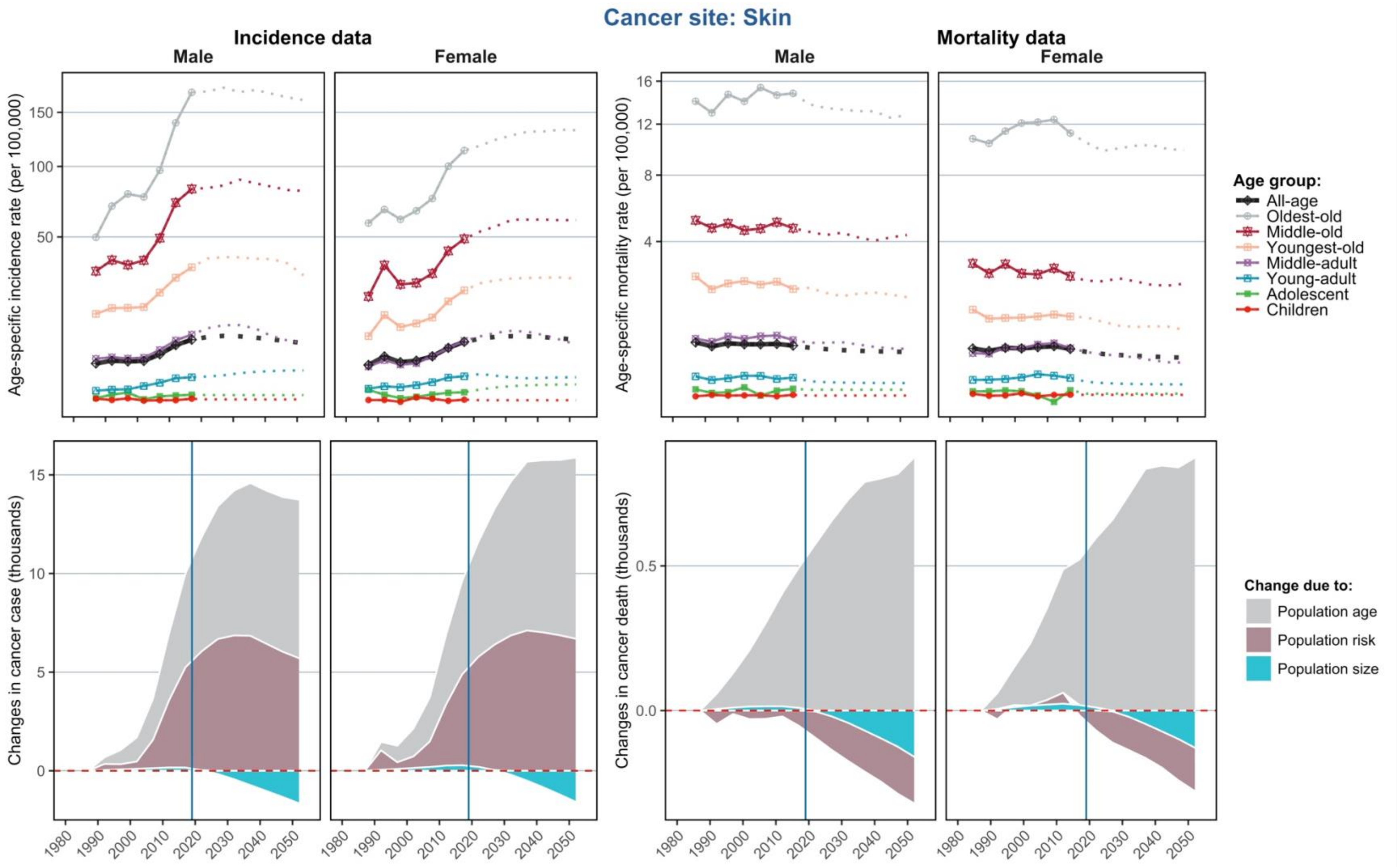

Figure S20. Projections of age-specific incidence and mortality rates and decomposed changes in new cases and deaths — Skin.

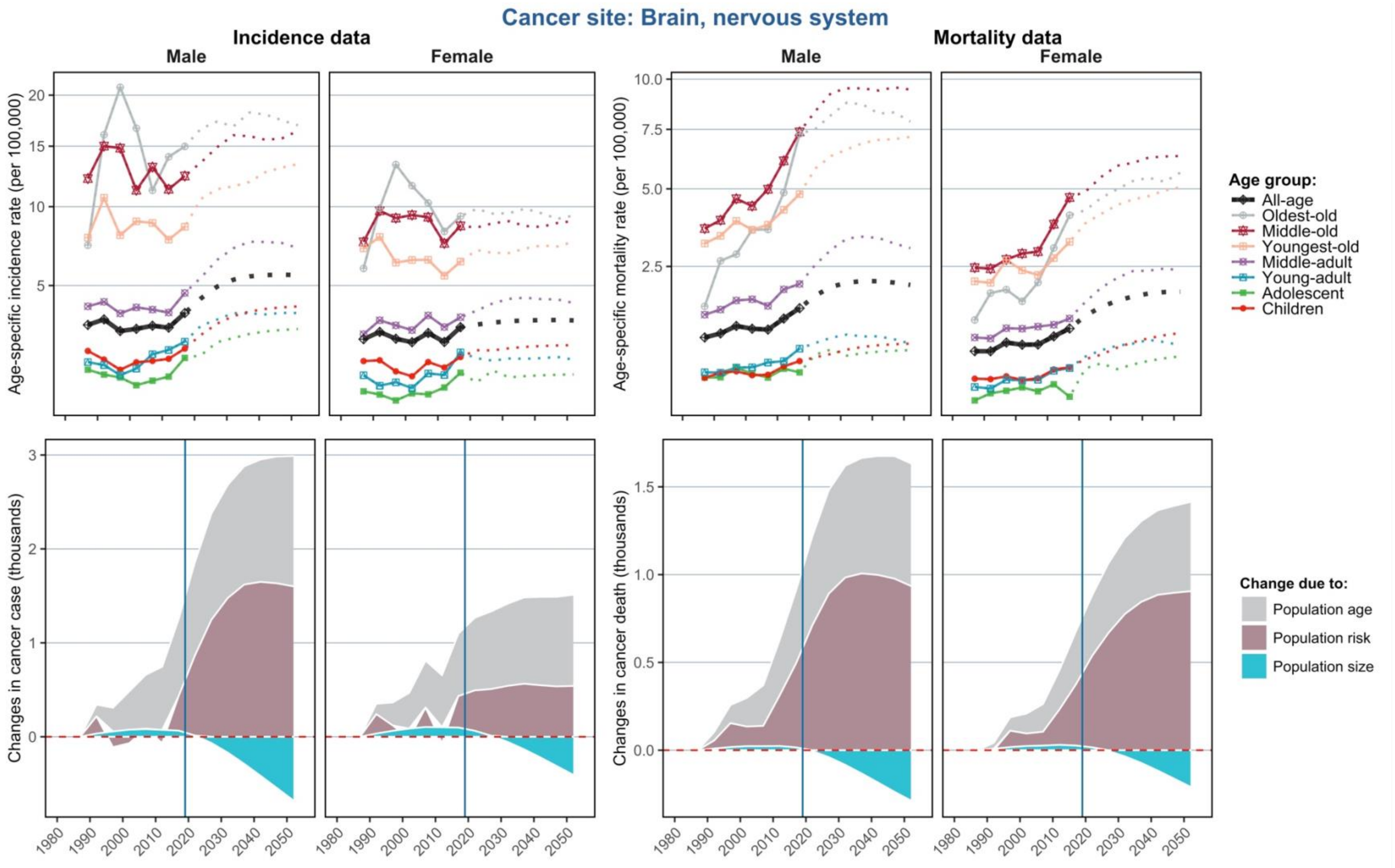

Figure S21. Projections of age-specific incidence and mortality rates and decomposed changes in new cases and deaths — Brain, nervous system.

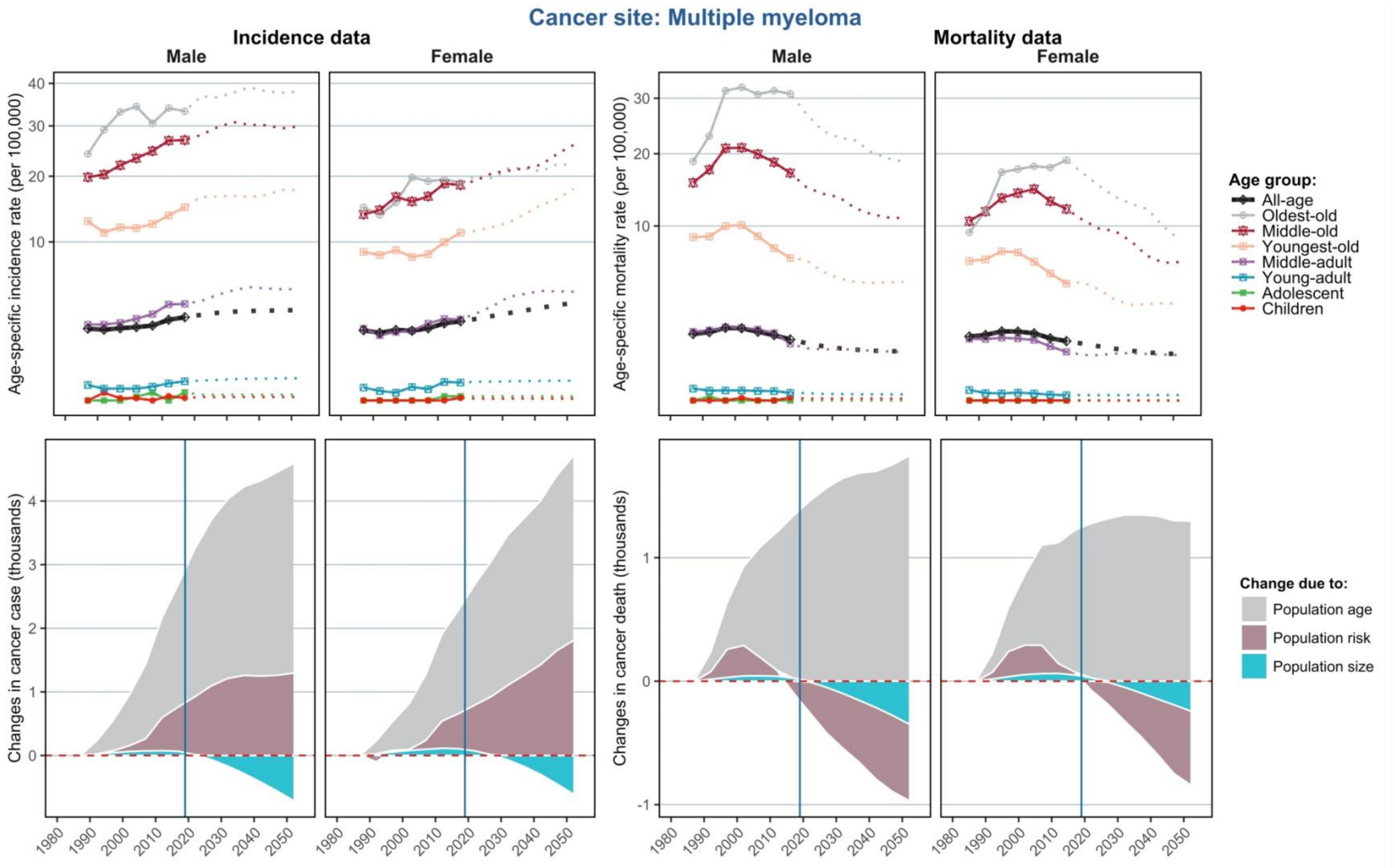

Figure S22. Projections of age-specific incidence and mortality rates and decomposed changes in new cases and deaths — Multiple myeloma.

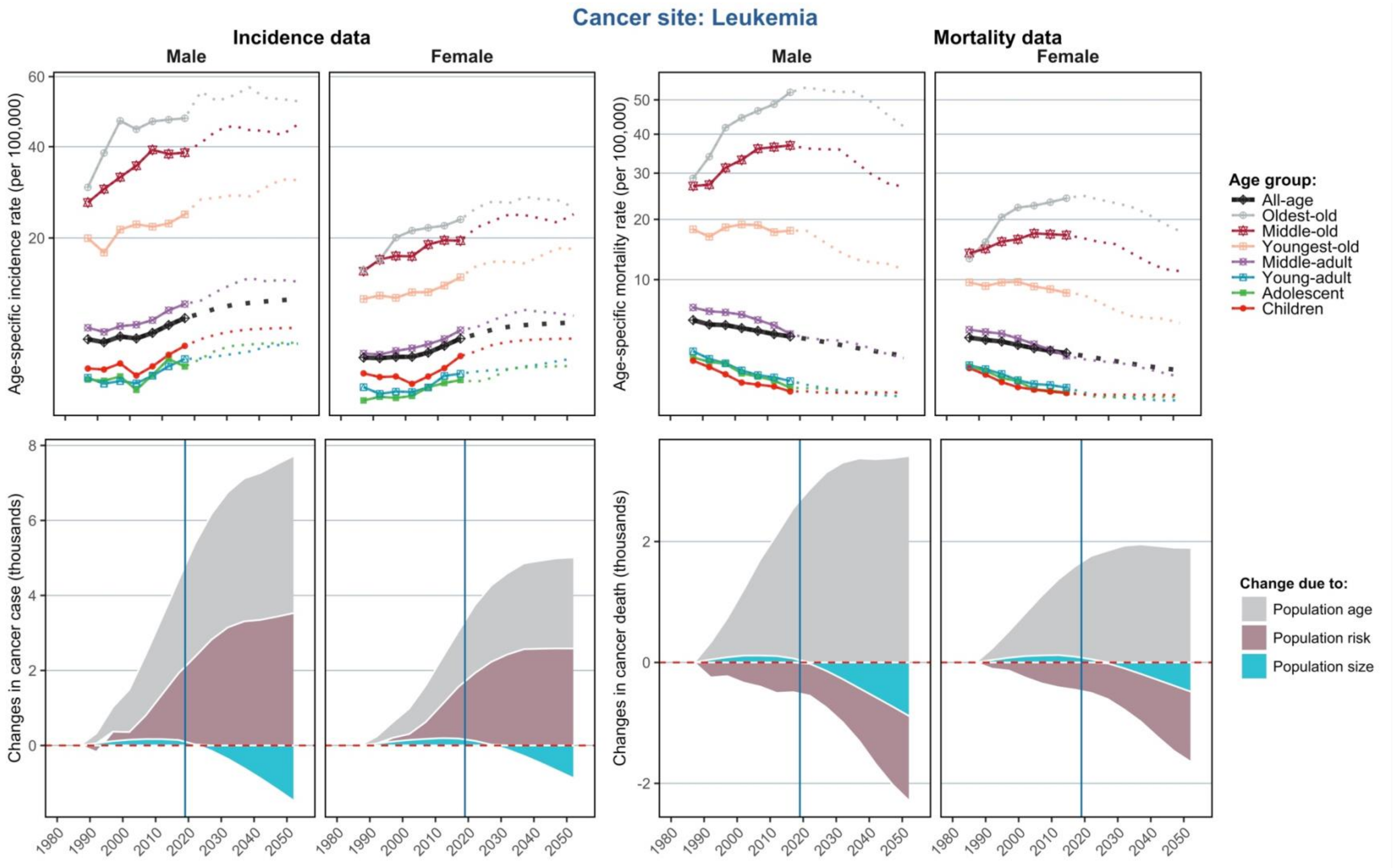

Figure S23. Projections of age-specific incidence and mortality rates and decomposed changes in new cases and deaths — Leukemia.

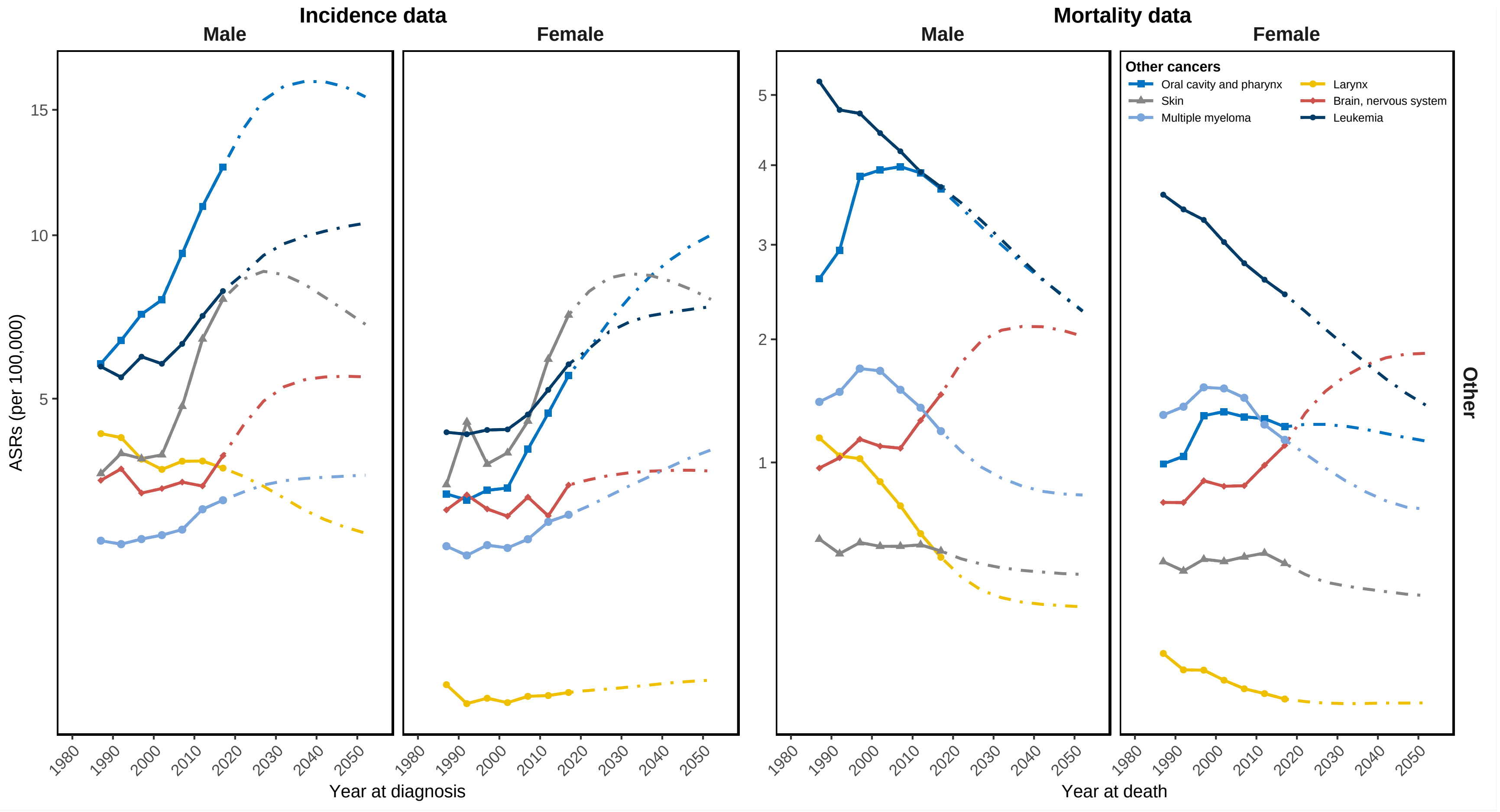

Figure S24. Projections of age-standardized incidence and mortality rates by genders for other cancer sites.

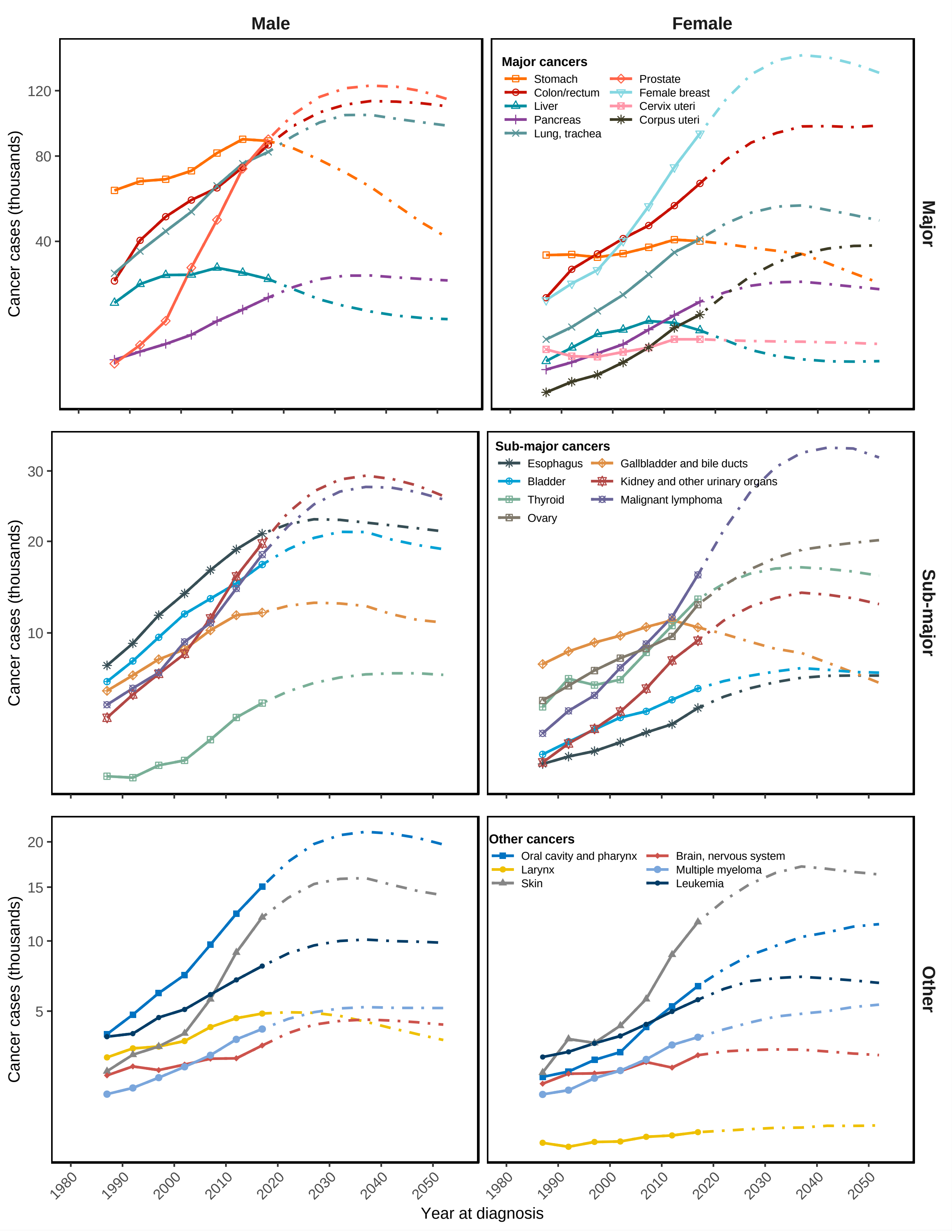

Figure S25. Projections of new cancer cases by genders for all cancer sites.

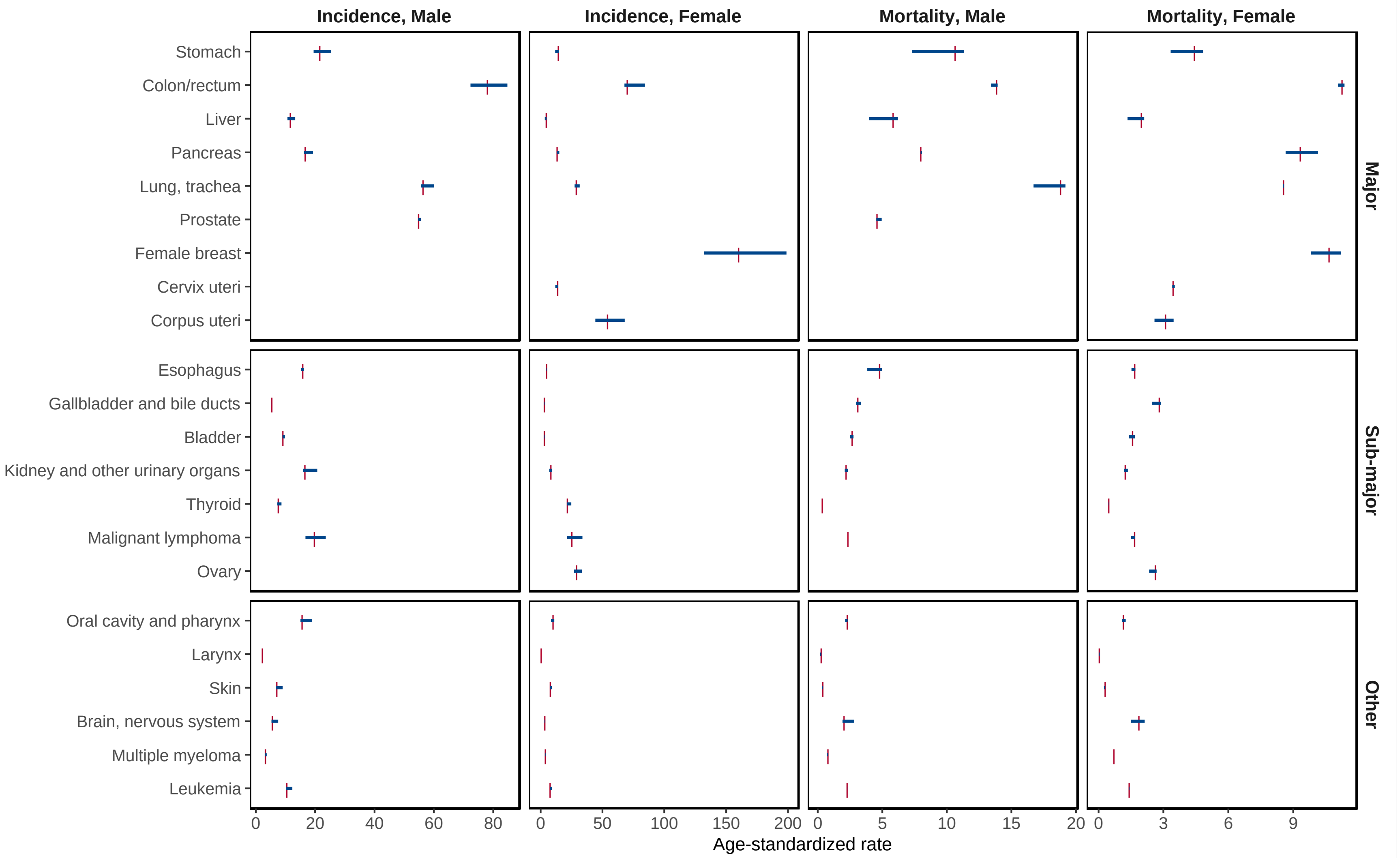

Figure S26. Sensitivity analysis of drift attenuation in ASR in 2050–2054.

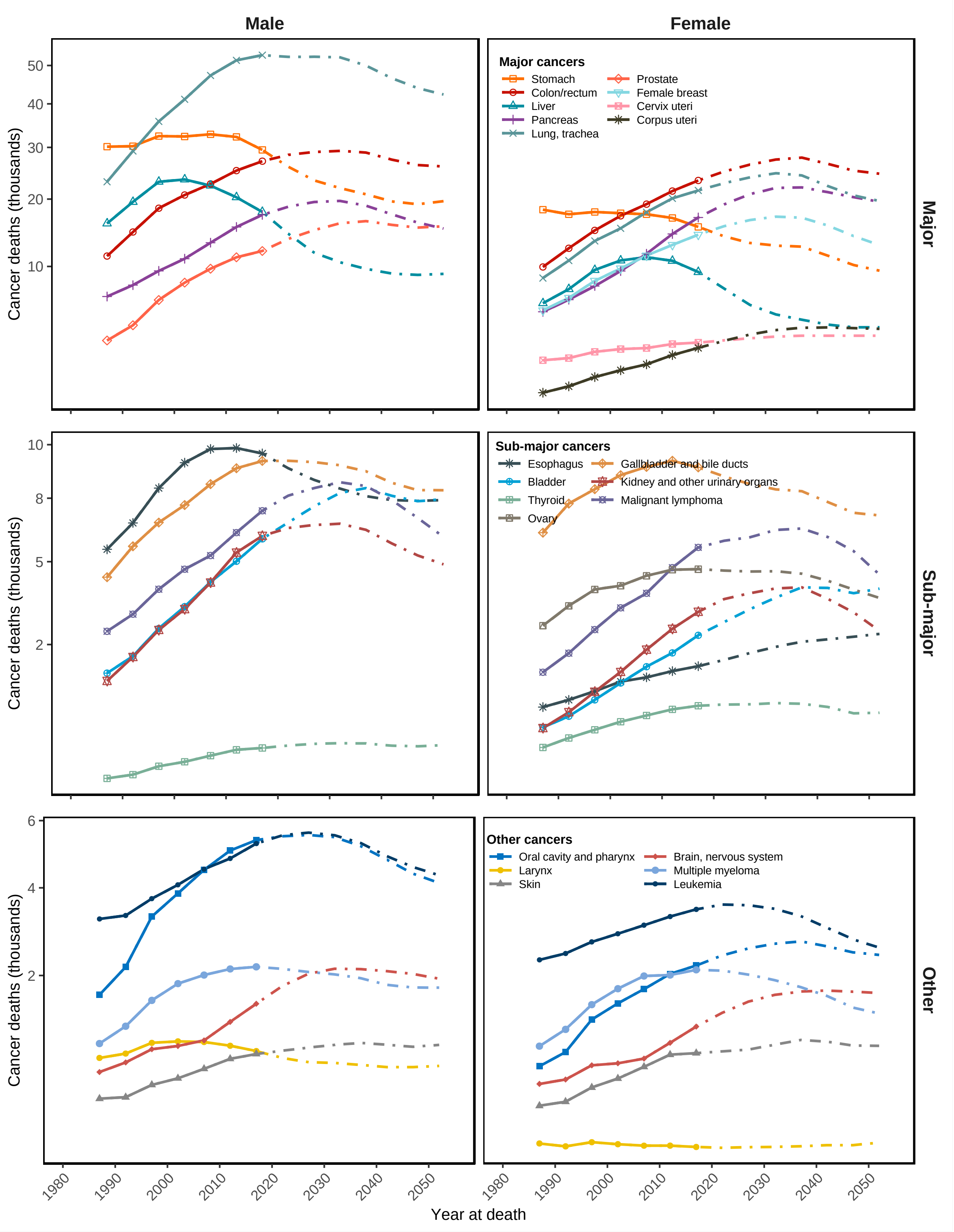

Figure S27. Projections of new cancer deaths by genders for all cancer sites.
